# Bacillus subtilis Histidine Kinase KinC Activates Biofilm Formation by Controlling Heterogeneity of Single-Cell Responses

**DOI:** 10.1128/mbio.01694-21

**Published:** 2022-01-11

**Authors:** Zhuo Chen, Priyanka Srivastava, Brenda Zarazúa-Osorio, Anuradha Marathe, Masaya Fujita, Oleg A. Igoshin

**Affiliations:** a Systems, Synthetic and Physical Biology Program, Rice University, Houston, Texas, USA; b Department of Biology and Biochemistry, University of Houston, Houston, Texas, USA; c Department of Bioengineering, Center for Theoretical Biological Physics, Department of Chemistry, and Department of Biosciences, Rice University, Houston, Texas, USA; The Ohio State University

**Keywords:** biofilm, modeling, intercellular heterogeneity, sporulation, systems biology

## Abstract

In Bacillus subtilis, biofilm and sporulation pathways are both controlled by a master regulator, Spo0A, which is activated by phosphorylation via a phosphorelay—a cascade of phosphotransfer reactions commencing with autophosphorylation of histidine kinases KinA, KinB, KinC, KinD, and KinE. However, it is unclear how the kinases, despite acting via the same regulator, Spo0A, differentially regulate downstream pathways, i.e., how KinA mainly activates sporulation genes and KinC mainly activates biofilm genes. In this work, we found that KinC also downregulates sporulation genes, suggesting that KinC has a negative effect on Spo0A activity. To explain this effect, with a mathematical model of the phosphorelay, we revealed that unlike KinA, which always activates Spo0A, KinC has distinct effects on Spo0A at different growth stages: during fast growth, KinC acts as a phosphate source and activates Spo0A, whereas during slow growth, KinC becomes a phosphate sink and contributes to decreasing Spo0A activity. However, under these conditions, KinC can still increase the population-mean biofilm matrix production activity. In a population, individual cells grow at different rates, and KinC would increase the Spo0A activity in the fast-growing cells but reduce the Spo0A activity in the slow-growing cells. This mechanism reduces single-cell heterogeneity of Spo0A activity, thereby increasing the fraction of cells that activate biofilm matrix production. Thus, KinC activates biofilm formation by controlling the fraction of cells activating biofilm gene expression.

## INTRODUCTION

Soil microbes are frequently exposed to environmental stress conditions, including nutrient starvation. To ensure survival, cells often sense environmental changes and respond by inducing a set of genes (1). Upon starvation, the soil bacterium Bacillus subtilis differentiates into distinct cell types. At the onset of starvation, a subset of B. subtilis cells lose their motility, form chains, and gain the ability to produce and secrete extracellular matrix (2, 3). As a result, biofilms—multicellular communities encased in the extracellular matrix—are formed (4). When starvation is prolonged, B. subtilis cells begin to form spores (5), which are metabolically inert and highly resistant to environmental stresses. In the starving community, growing, biofilm-forming, and sporulating cell types coexist (6, 7), but the mechanisms controlling this heterogeneity are not fully uncovered.

Both biofilm matrix production and sporulation are triggered by the activation of Spo0A, a master regulatory transcription factor (8, 9). The phosphorylated form of Spo0A (Spo0A∼P) acts as a transcriptional regulator that directly and indirectly controls sporulation genes (10, 11), and the threshold amount of Spo0A∼P triggers sporulation (10, 12, 13). Moreover, Spo0A∼P indirectly activates genes related to biofilm matrix production via the SinI-SinR-SlrR regulatory network. Spo0A∼P activates the expression of SinI, which in turn sequesters and thereby inactivates the transcriptional regulator SinR (14, 15). SinR represses the expression of SlrR, which can also sequester and inactivate SinR (16, 17). Inactivation of SinR results in the derepression of a set of genes and operons controlling biofilm formation, including the *tapA* (formerly named *yqxM*)*-sipW-tasA* operon, encoding proteins required for proper extracellular matrix formation (18).

The phosphorylation of Spo0A is achieved via a four-component His-Asp-His-Asp phosphotransfer cascade termed phosphorelay (19). At the top of the cascade, there are at least five histidine kinases (KinA, KinB, KinC, KinD, and KinE) that are autophosphorylated under different conditions (20, 21). Thereafter, the phosphate (phosphoryl group) from each of the phosphorylated kinases is transferred to two intermediate phosphotransferases Spo0F and Spo0B, in this order, and eventually to Spo0A (19). The concentration of Spo0A∼P is further fine-tuned by the phosphatase Spo0E, which dephosphorylates Spo0A∼P (22).

Intriguingly, despite acting via the same biochemical mechanism and via the same master regulator, different kinases play distinct roles in the regulation of cell differentiation. KinA appears to be primarily responsible for sporulation upon starvation (21). KinA autophosphorylation is inhibited by Sda, which acts as a checkpoint protein to ensure proper sporulation (23–25). Our study showed that the slowdown of growth leads to an increase in cellular KinA concentration, which eventually results in the initiation of sporulation (26). On the other hand, KinC mainly controls biofilm formation (27). How different kinases activate the same transcription factor but lead to distinct cell fates is still unclear.

In this work, combining experimental data with mathematical modeling, we explain how KinA and KinC interplay in the regulation of biofilm matrix production and sporulation. Our experiments showed that deletion of *kinC* increases sporulation gene expression, suggesting that KinC negatively affects cellular Spo0A∼P concentration. To explain this effect, we used a mathematical model of phosphorelay. The model predicted that, when KinA is active, KinC reduces Spo0A∼P concentration by acting as a phosphate sink. However, this prediction seemingly contradicts the positive effect of KinC on biofilm gene expression under the same conditions. To reconcile the conflicting roles of KinC in two gene expression programs, we investigated how KinC affects heterogeneity in single-cell gene expression. As predicted by the model, the presence of KinC reduces the cell-to-cell heterogeneity of Spo0A∼P concentrations. As a result, more cells will reach sufficient Spo0A∼P concentration to activate biofilm gene expression. Thus, KinC positively regulates biofilm formation by increasing the fraction of cells that can activate biofilm matrix production.

## RESULTS

### Different effects of KinC and KinA on matrix production and sporulation.

To understand the roles of KinA and KinC in the regulation of Spo0A and cell fate decisions at the population level, we measured the activities of two different Spo0A-controlled promoters, *tapA* and *spoIIG* (see Materials and Methods for details). The *tapA* operon encodes proteins required for matrix production (18, 28). Its expression is indirectly activated by Spo0A∼P at a low-concentration threshold (10). The *spoIIG* operon encodes the mother cell-specific pro-σ^E^ and its processing enzyme (29). Under sporulation conditions, it is directly controlled by Spo0A∼P at a high threshold (10, 30). For comparison, we also investigated the effects of *kinB* deletion and *kinD* deletion on *tapA* expression. The results indicate that the deletion of *kinB* has an effect very similar to that of *kinA* deletion (Fig. S1A), suggesting that these kinases can be lumped together in our models. Furthermore, under our conditions, the effect of *kinD* is minor (Fig. S1B).

10.1128/mBio.01694-21.1 FIG S1 Effect of KinA, KinB, KinC and KinD on *tapA* expression. Measured dynamics of *PtapA* activities are shown for WT, Δ*kinA*, Δ*kinB*, and Δ*kinC* strains (A) and WT and Δ*kinD* strains (B). Download FIG S1, PDF file, 0.4 MB. Copyright © 2022 Chen et al. 2022 Chen et al. https://creativecommons.org/licenses/by/4.0/ This content is distributed under the terms of the Creative Commons Attribution 4.0 International license.

**FIG 1:**
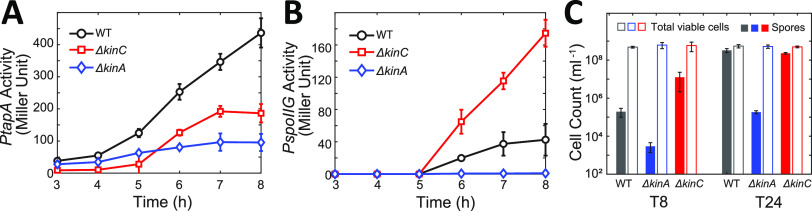
Effect of KinA and KinC on *PtapA*/*PspoIIG* activity and sporulation. (A and B) The dynamics of β-galactosidase activities from *PtapA-lacZ* (A) and *PspoIIG-lacZ* (B) in different strains (WT, Δ*kinC*, and Δ*kinA*). The strains were cultured in liquid MSgg medium. The samples were collected at the indicated times after culture. The mean activities of at least three independent experiments are shown with standard deviations. (C) Counts of spores and total viable cells of different strains at 8 and 24 h (T8 and T24, respectively) of culture in liquid MSgg medium. The mean counts of at least four independent experiments are shown with standard deviations.

The dynamics of *PtapA* activity suggest that KinA and KinC are active at different stages of growth in liquid MSgg medium conditions (see Materials and Methods for details). In the Δ*kinA* strain, the *PtapA* activity was only slightly lower than in the wild-type (WT) strain at early times (Fig. 1A, 3 to 5 h of culture). However, after 5 h of culture, the *PtapA* activity stopped increasing and became much lower in the Δ*kinA* strain than in the WT strain. Thus, KinA is active mainly during later times of starvation, when cell growth slows down, which is in agreement with published results (21, 26). In contrast, in the Δ*kinC* strain, the *PtapA* activity was significantly lower than in WT cells at all times (Fig. 1A). These results indicate that KinC-dependent activation of Spo0A increases matrix gene expression throughout the experimental time course.

In contrast, our results indicate a different effect of KinC on sporulation gene expression. As Fig. 1B shows, at early times (3 to 5 h of culture), *PspoIIG* activity remained very low in all the strains. At later times (after 5 h of culture), the *PspoIIG* activity increased in the WT and the Δ*kinC* strains, but the Δ*kinC* strain showed higher activity than the WT strain (Fig. 1B). In comparison, no *PspoIIG* activity was detected in the Δ*kinA* strain (Fig. 1B).

To further examine the role of KinC in the regulation of sporulation, we measured the sporulation efficiencies of WT and Δ*kinC* cells grown in MSgg medium. To this end, we counted the number of spores and the total number of viable cells (Fig. 1C; also, see Materials and Methods for details). We found that the Δ*kinC* strain showed early onset of sporulation compared with the WT strain in MSgg (Fig. 1C, T8). After 24 h of culture, many of the WT cells would form spores, so the deletion of *kinC* cannot further increase the spore counts (Fig. 1C, T24). These results indicate that the deletion of *kinC* accelerates sporulation. In contrast, the sporulation in the Δ*kinA* strain is greatly diminished (Fig. 1C), consistent with previous studies (21). No significant effect of deletion on the total number of viable cells was observed. Taken together, our results suggest that KinC negatively affects Spo0A∼P levels after 5 h of culture, keeping it below the threshold required for sporulation.

### A kinetic model of the phosphorelay provides an explanation of the distinct roles of KinC in the regulation of Spo0A∼P.

**FIG 2:**
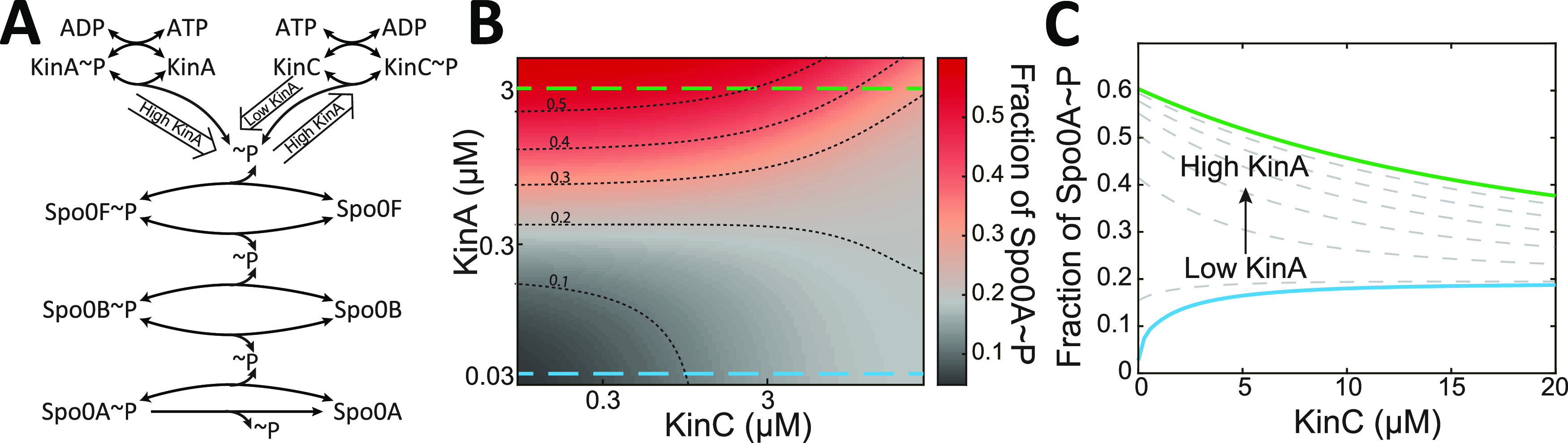
Mathematical model explaining the different effects of KinC on Spo0A∼P concentrations. (A) Kinetic scheme of the phosphorelay network explicitly showing two phosphorylation pathways by two kinases. The hollow arrows indicate the direction of phosphate flux: When KinA concentrations are low, KinC preferentially transfers the phosphate to Spo0F and maintains an intermediate Spo0A∼P level. At high KinA concentrations, the direction of phosphate flux is flipped, and KinC acts as a phosphate sink. (B) Predicted fraction of Spo0A∼P as a function of KinA and KinC concentrations. The black dashed lines are the contour lines of the Spo0A∼P fraction. The green and blue dashed lines mark the KinA concentrations corresponding to the green and blue curves in panel C. (C) The predicted fraction of Spo0A∼P changes with KinC concentrations in different manners under different concentrations of KinA. When KinA concentration is low (0.03 μM; green curve), the fraction of Spo0A∼P increases with KinC concentrations. At high KinA concentration (3 μM, blue curve), higher KinC concentrations would result in lower fractions of Spo0A∼P. The dashed lines show the Spo0A∼P fractions at intermediate KinA concentrations (0.1 μM, 0.4 μM, 0.8 μM, 1.2 μM, 1.6 μM, and 2 μM).

To further explore the roles of KinA and KinC in the regulation of Spo0A∼P, we employed a detailed mathematical model of phosphorelay (Fig. 2A; also, see Materials and Methods for details). The model was extended from earlier published models from our group which were calibrated using experimental data (12, 13, 26, 31, 32). Given that KinC is not as efficient as KinA in phosphotransfer to Spo0F (33), we assigned different kinetic parameters to KinA and KinC (see Materials and Methods for details). Notably, we modeled all of the phosphotransfer reactions as reversible (Fig. 2A) based on the prior *in vitro* experiments (20, 34). The model allowed us to investigate how KinA and KinC together regulate Spo0A∼P.

The model predicted how the fraction of Spo0A∼P (the concentration of Spo0A∼P divided by the concentration of total Spo0A) changes as a function of KinA and KinC concentrations. Due to different kinetic parameters, the phosphate flux from KinA to Spo0F is larger than that from KinC to Spo0F. As Fig. 2B shows, the model predicted that Spo0A∼P fraction is low when both KinA and KinC concentrations are low. The increase in either KinA or KinC leads to the increase in Spo0A∼P fraction but to different extents; the increase in KinA concentration leads to a much higher phosphorylated fraction (Fig. 2B). These results reveal that by itself KinA generates high levels of Spo0A∼P while KinC by itself maintains intermediate levels.

Furthermore, this model predicted the distinct effects of KinC on Spo0A∼P at different KinA concentrations. When KinA concentrations are high (Fig. 2B), the increase in KinC concentration would decrease rather than increase the fraction of phosphorylated Spo0A∼P. The distinct effects of KinC on Spo0A∼P are further illustrated in Fig. 2C. When KinA concentrations are low, the autophosphorylation of KinC is the major source of phosphate, so the steady-state Spo0A∼P levels would increase when KinC concentration increases. However, at high concentrations of KinA, Spo0A∼P levels would be negatively correlated with KinC concentration. In that case, KinC acts as a phosphate sink, and the direction of the phosphotransfer reaction conducted by KinC would be shifted in the reverse reaction from Spo0F∼P to KinC (Fig. 2A, hollow arrow). This prediction of the model is robust to the uncertainty of the parameter values (Fig. S6; also, see Materials and Methods for details).

10.1128/mBio.01694-21.6 FIG S6 Robustness of the negative effect of KinC in the phosphorelay to the uncertainty of model parameters. (A) Representative points to test the conclusion of the model. We selected four representative points, representing low KinA and low KinC, high KinA and low KinC, low KinA and high KinC, and high KinA and high KinC. The fractions of Spo0A∼P under those conditions were labeled as *f*_1_ ∼ *f*_4_. (B) Robustness of the phosphorelay model. The fractions *f*_1_/*f*_2_ and *f*_3_/*f*_4_ were calculated for 1,000 different parameter combinations. All of the parameters are sampled from a uniform distribution on the interval [1/3 *p_i_*, 3 *p_i_*], where *p_i_* is the original value of the *i*th parameter. The fractions *f*_1_/*f*_2_ and *f*_3_/*f*_4_ for the original parameters are shown as a red point. For all points, the value *f*_1_/*f*_2_ is smaller than 1; for 976/1,000 points, the value *f*_3_/*f*_4_ is smaller than 1 (to the left of the red dashed line). The results indicate that our conclusion, that KinC positively affects Spo0A∼P at low levels of KinA (*f*_1_ < *f*_2_) and negatively affects Spo0A∼P at high levels of KinA (*f*_3_ < *f*_4_), is robust against the fluctuation of parameters. Download FIG S6, PDF file, 0.6 MB. Copyright © 2022 Chen et al. 2022 Chen et al. https://creativecommons.org/licenses/by/4.0/ This content is distributed under the terms of the Creative Commons Attribution 4.0 International license.

### The model provides an explanation of how KinC differently regulates Spo0A∼P at different growth stages.

To assess if the distinct roles of KinC for Spo0A∼P (Fig. 2A and B) are sufficient to explain the observed dynamics of *PtapA* and *PspoIIG* activity (Fig. 1A and B), we need to model how the concentrations of KinA and KinC change with time. Recent experimental and theoretical studies reveal that the slowdown of cell growth increases the cellular concentrations of KinA, leading to the pulsatile increase in Spo0A level and activity (26, 31, 35–37). Although multiple mechanisms of nutrient or starvation sensing could be employed by cells, using the cell growth rate as an indirect measure of the sensory system is particularly appealing, since it bypasses the need for any dedicated metabolite sensing systems (26, 27, 38). Thus, we assumed that the decrease of growth rate drives the increase in Spo0A∼P concentrations via the increase in the concentration of KinA and KinC proteins.

A slowdown of growth leads to the accumulation of stable proteins (39). In our model, several processes contribute to this accumulation (see Materials and Methods for details). First, the growth rate determines the protein dilution rate. For stable proteins, the effect of dilution is stronger than degradation; therefore, a lower growth rate leads to slower dilution and higher concentration (39). Second, slow-growing cells have smaller cell volumes (26), which also leads to higher cellular concentrations of proteins with the same copy number. For simplicity, the transcription rate and translation rate are assumed to be independent of growth rate. The model successfully explained the effect of growth rate on the concentration of stable proteins (26). To incorporate slowdown of growth into our model, we measured the growth curve during the starvation and fit a simple growth dynamics model to estimate cell growth rate at different times (see Materials and Methods and Fig. S3A for details). Assuming that the biochemical reactions are faster than the change of growth rate, we can use quasi-steady-state approximation to predict the steady-state concentration of KinA and KinC at different times.

10.1128/mBio.01694-21.3 FIG S3 Model of growth dynamics and heterogeneity. (A) Growth curve and the dynamics of growth rate. The circles show the measured cell density in OD values. The red dashed line shows the dynamics of the growth rate given by the model of growth dynamics (see Materials and Methods for details) that was fitted to the experimental data. (B) The assumed distribution of cell growth rate at T6. (C) Experimentally measured growth curves of different strains. Download FIG S3, PDF file, 0.4 MB. Copyright © 2022 Chen et al. 2022 Chen et al. https://creativecommons.org/licenses/by/4.0/ This content is distributed under the terms of the Creative Commons Attribution 4.0 International license.

**FIG 3:**
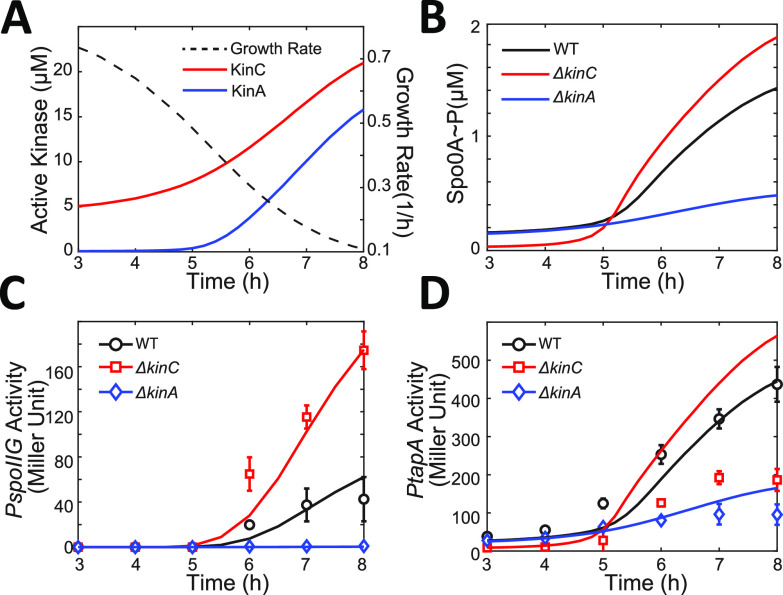
Effect of growth rate changes explains the roles of KinC at different growth stages. (A) Predicted dynamics of the concentration of active KinA/KinC (solid lines, left *y* axis) and growth rate (dashed line, right *y* axis). The growth rate dynamics are calculated with a model that is fitted to the experimental data (see Materials and Methods for details). (B) Predicted dynamics of Spo0A∼P concentration in WT, Δ*kinC*, and Δ*kinA* strains. (C and D) Predicted dynamics of *PspoIIG* (C) and *tapA* (D) activity in WT, Δ*kinC*, and Δ*kinA* strains. The solid line represents a model fit for the *PspoIIG* activity dynamics. The experimental data presented in Fig. 1 are rearranged and presented as different markers.

Sda, an inhibitor of KinA, also affects the growth rate dependency of KinA activity. Sda inhibits KinA by binding to its autokinase domain (23, 24). It is also known that growing cells with actively replicating DNA produces increasing concentrations of Sda, leading to inhibition of KinA and thus inhibiting entry into sporulation (40). Our experiment showed that the deletion of *sda* could restore *tapA* expression at early times in the Δ*kinC* strain (Fig. S2A). The result suggests that Sda also affects matrix production by inhibiting KinA at the early stages of starvation. Therefore, we introduced Sda into the model. Notably, Sda is subject to rapid degradation *in vivo* (25), whereas KinA and KinC were assumed to be stable proteins. As a result, the slowdown of growth would increase KinA concentration but would only slightly affect Sda concentration (Fig. S2B). While Sda could also inhibit KinC, this inhibition is significantly less effective than that of KinA (23). Thus, the Sda inhibition on KinA makes the concentration of active KinA (free from Sda) more sensitive to growth rate than KinC (Fig. 3A). As the growth rate decreases, the concentration of KinC gradually increases due to the decrease of dilution rate and cell volume. As for KinA, at early times (before 5 h), the concentration of active KinA is very low because of the excess of Sda. At later times, KinA concentration increases but Sda concentration does not change much, so Sda can no longer fully inhibit KinA (Fig. S2C). As a result, the concentration of active KinA rapidly increases after 5 h (Fig. 3A).

10.1128/mBio.01694-21.2 FIG S2 Sda plays an important role in the regulation of Spo0A∼P. (A) Measured dynamics of *PtapA* activity in WT, Δ*kinC*, Δ*sda*, and Δ*kinC* Δ*sda* strains. The deletion of *sda* significantly promotes *tapA* expression in both WT and Δ*kinC* backgrounds throughout the time range. This result indicates that Sda inhibits KinA and preventing it from activating *tapA* expression. (B) Predicted total concentrations of Sda and KinA as functions of the growth rate. (C) Predicted dynamics of the total concentrations of Sda and KinA. Download FIG S2, PDF file, 0.4 MB. Copyright © 2022 Chen et al. 2022 Chen et al. https://creativecommons.org/licenses/by/4.0/ This content is distributed under the terms of the Creative Commons Attribution 4.0 International license.

Using the dynamics of concentrations of active KinA and KinC as inputs, our model explains how KinA and KinC affect the dynamics of Spo0A∼P. At early times, KinA is inhibited by Sda, and KinC is the main phosphate source involved in the phosphorelay. Thus, the deletion of *kinC* reduces Spo0A∼P concentrations before ∼5 h, whereas the deletion of *kinA* has a minor effect (Fig. 3B). At later times (after ∼5 h), the concentration of active KinA increases in the starving WT cells, and KinA become the major phosphate source, so the deletion of *kinA* would significantly reduce the Spo0A∼P concentrations. In that case, KinC acts as a phosphate sink and negatively regulates Spo0A∼P, so the deletion of *kinC* results in higher Spo0A∼P concentrations (Fig. 3B).

To see if the predicted Spo0A∼P concentrations can explain observed expression patterns of the downstream genes, we first assumed that *tapA* and *spoIIG* expression are determined by Spo0A∼P with monotonic phenomenological functions (see Materials and Methods for details; the unknown parameters were optimized to fit the experimental data). The results successfully explain the effect of KinA and KinC on the dynamics of *spoIIG* expression (Fig. 3C). Expression of *spoIIG* is known to be directly activated by Spo0A∼P with a high threshold (10). As shown in Fig. 3C, before ∼5 h, the *PspoIIG* activity is low in all three strains, and the positive effect of KinC on Spo0A∼P does not significantly affect the expression of *spoIIG*. After ∼5 h, in the WT strain, due to the accumulation of KinA, the Spo0A∼P concentrations increase and become high enough to trigger *spoIIG* expression. Thus, the deletion of *kinA* would eliminate *spoIIG* expression, while the deletion of *kinC* raises Spo0A∼P concentrations, leading to higher *spoIIG* expression. The model matched the experimental data well (Fig. 3C), supporting our proposed model that KinC negatively regulates Spo0A∼P by acting as a phosphate sink.

In contrast, the model is unable to fully explain the observed patterns of *tapA* expression. The fitted model simulations were in good agreement with the experimental data of *PtapA* activity dynamics in the WT and Δ*kinA* strains but not the Δ*kinC* strain (Fig. 3D). The model predicted that the *PtapA* activity would be higher in the Δ*kinC* strain than in the WT strain because of the higher Spo0A∼P concentrations after ∼5 h, whereas experimental data showed lower *PtapA* activity in the Δ*kinC* strain than the WT strain over the whole time range (Fig. 3D). Thus, the model assuming monotonic (Hill function) activation of gene expression on Spo0A∼P is not able to fully explain the effect of Δ*kinC* on *tapA*.

One possible way to resolve this contradiction is to assume that Spo0A∼P has different effects on *tapA* expression depending on the concentrations of Spo0A∼P, i.e., assume that *tapA* is repressed by high concentrations of Spo0A∼P, as suggested by Chai et al. (41). However, when included in our model, this high-concentration repression mechanism would lead to nonmonotonic dynamics of *PtapA* activity in the Δ*kinC* strain (Fig. S4A), which was not observed in the experiment (Fig. 1A). To further test the effect of high concentrations of Spo0A∼P on *PtapA* activity, we measured *PtapA* activity in a strain with *kinA* overexpressed using an IPTG (isopropyl-β-d-thiogalactopyranoside)-inducible promoter (37, 42). The results indicate that high Spo0A∼P concentrations generated by overexpressed KinA did not repress *PtapA* activity. Instead, the overexpression of KinA resulted in much higher *PtapA* activity than in the WT strain (Fig. S4B). Thus, the results indicate that the reduction of *tapA* expression in the Δ*kinC* strain is not due to the increase in Spo0A∼P concentrations.

10.1128/mBio.01694-21.4 FIG S4 The repression of *tapA* expression by high Spo0A∼P levels cannot explain the *tapA* promoter activity dynamics in Δ*kinC* strain. (A) The predicted dynamics of *PtapA* activity in different strains assuming high Spo0A∼P levels could repress *tapA* expression. The model predicts that *tapA* promoter activity of Δ*kinC* strain would rise rapidly at ∼5 h, become higher than that of the WT, and then decrease after ∼6.5 h. These dynamics are not observed in the experimental data. (B) High Spo0A∼P levels would not repress *tapA* expression. The dynamics of *PtapA* activity in WT and *kinA*-overexpressed strains were measured and plotted. If high levels of Spo0A∼P could repress *tapA* expression at late times in the Δ*kinC* strain, the overexpression of *kinA* should also repress *tapA* expression. On the contrary, the overexpression of *kinA* dramatically increases *tapA* expression. Download FIG S4, PDF file, 0.4 MB. Copyright © 2022 Chen et al. 2022 Chen et al. https://creativecommons.org/licenses/by/4.0/ This content is distributed under the terms of the Creative Commons Attribution 4.0 International license.

### KinC reduces the cell-to-cell heterogeneity of Spo0A∼P concentrations.

**FIG 4:**
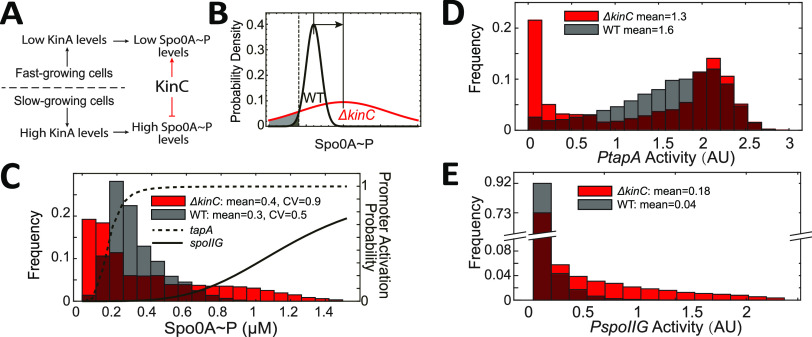
Effect of KinC on the heterogeneity of Spo0A∼P levels in individual cells. (A) KinC reduces the heterogeneity of Spo0A levels in individual cells. In fast-growing cells, KinA levels are low, so KinC induces intermediate Spo0A∼P levels. In slow-growing cells, KinA accumulates and induces high levels of Spo0A∼P, while KinC reduces Spo0A∼P levels. (B) A higher mean Spo0A∼P level does not guarantee a higher mean *PtapA* activity. For a toy model, we assume that the Spo0A∼P levels in WT and Δ*kinC* strains follow normal distributions. The mean and variance of Spo0A∼P level are both higher in the Δ*kinC* strain. The gray area indicates the fraction of cells not expressing *tapA*. The dashed line shows the threshold level of Spo0A∼P to activate *tapA*. (C) Predicted distribution of Spo0A∼P level in Δ*kinC* and WT strains. The solid and dashed lines corresponding to the right *y* axis show how activities of *PspoIIG* and *PtapA,* respectively, depend on Spo0A∼P level. (D and E) Predicted distributions of *PtapA* (D) and *PspoIIG* (E) activity in Δ*kinC* and WT strains.

The results thus far indicate that some features of the Spo0A regulatory network were not captured by the model. One of those features is cell-to-cell heterogeneity in the Spo0A∼P concentrations and the resulting heterogeneity of downstream gene expression. A study from our group demonstrated that the noise in growth rate determines the distribution of single-cell Spo0A∼P concentrations and explains the heterogeneity of sporulation in single cells (26). We therefore hypothesized that the noise in Spo0A∼P concentrations originated from the growth rate fluctuations and that KinC can affect the noise propagation from growth rate to Spo0A∼P. The rationale for this hypothesis is shown in Fig. 4A. Based on the results in Fig. 3B, we assumed that KinC would affect cells at different growth stages differently due to different concentrations of active KinA. In the same population, KinA concentrations are low for faster-growing cells, and in these cells, KinC contributes to the increase in Spo0A∼P concentrations. On the other hand, in the slow-growing individual cells, KinA concentrations are high. In that case, KinC acts as a phosphate sink and reduces Spo0A∼P concentration. With this “rob the rich and help the poor” mechanism, KinC reduces the cell-to-cell heterogeneity in the Spo0A∼P concentrations.

The above-described effect can lead to counterintuitive effects on downstream gene expression. As Fig. 4B shows, if both mean and variance of Spo0A∼P levels are increased in the Δ*kinC* strain, it is possible that the fraction of cells with Spo0A∼P levels lower than a certain threshold (Fig. 4B, gray area) also increases. Therefore, we hypothesized that the observed population-level decrease of *tapA* expression in the Δ*kinC* strain is due to the increase in the fraction of cells that failed to activate *tapA* expression.

To quantitatively assess the feasibility of the mechanism hypothesized as above, we constructed a simple model to test how growth rate heterogeneity affects the distribution of *PtapA* activity in different strains. In this model, we assumed that the noise in growth rate fully determines the noise in Spo0A∼P concentration and finally determines the heterogeneity of *tapA* expression. It is known that the heterogeneity of individual cell generation time of B. subtilis during balanced growth could be approximated as normally distributed (43). Here, we assumed that the cell generation time during starvation also follows normal distribution but with the mean value changing with starvation duration. The cell generation times were sampled and then the growth rates were calculated accordingly (Fig. S3B; also, see Materials and Methods for details). For each growth rate, using the same model as for Fig. 3B, we calculated the steady-state Spo0A∼P concentrations in individual cells in a culture population. The results indicate that KinC contributes to the decrease in the mean and the variance of Spo0A∼P concentrations in individual cells (Fig. 4C).

Using Hill functions to model the expression of *tapA* and *spoIIG* as functions of Spo0A∼P (Fig. 4C, solid and dashed lines), we can predict the effect of KinC deletion on *tapA* and *spoIIG* expression. A low concentration of Spo0A∼P is enough to activate *tapA* expression (Fig. 4C, dashed line). In the WT strain, most of the cells maintained certain Spo0A∼P concentrations (>0.2 μM), which is sufficient to activate *PtapA*. Thus, most of the WT cells expressed *tapA*. In contrast, in the Δ*kinC* strain, near 40% of cells showed very low Spo0A∼P concentrations (<0.2 μM) (Fig. 4C), thus they did not express *tapA* (Fig. 4D). As a result, lower mean *PtapA* activity was predicted in the Δ*kinC* strain than in the WT strain (Fig. 4D). As to *spoIIG*, since the expression of *spoIIG* needs a higher concentration of Spo0A∼P (Fig. 4C, solid line), the mean *PspoIIG* activity was predicted to be higher in Δ*kinC* strain because of higher Spo0A∼P concentrations (Fig. 4E).

Therefore, the model qualitatively explains the paradox that the Δ*kinC* strain has higher Spo0A∼P concentrations but lower *PtapA* activity. For those fast-growing cells, the deletion of *kinC* results in lower Spo0A∼P concentrations, so their *PtapA* activity is reduced. For slow-growing cells, Spo0A∼P concentrations are high enough to saturate *PtapA*. Though the deletion of *kinC* leads to higher Spo0A∼P concentrations, the *PtapA* activity did not further increase in slow-growing cells. As a result, despite higher mean Spo0A∼P concentrations, the Δ*kinC* strain displays lower mean *PtapA* activity than the WT strain (Fig. 4D).

### KinC mainly affects the fraction of *tapA*-expressing cells instead of the *tapA* expression levels.

**FIG 5:**
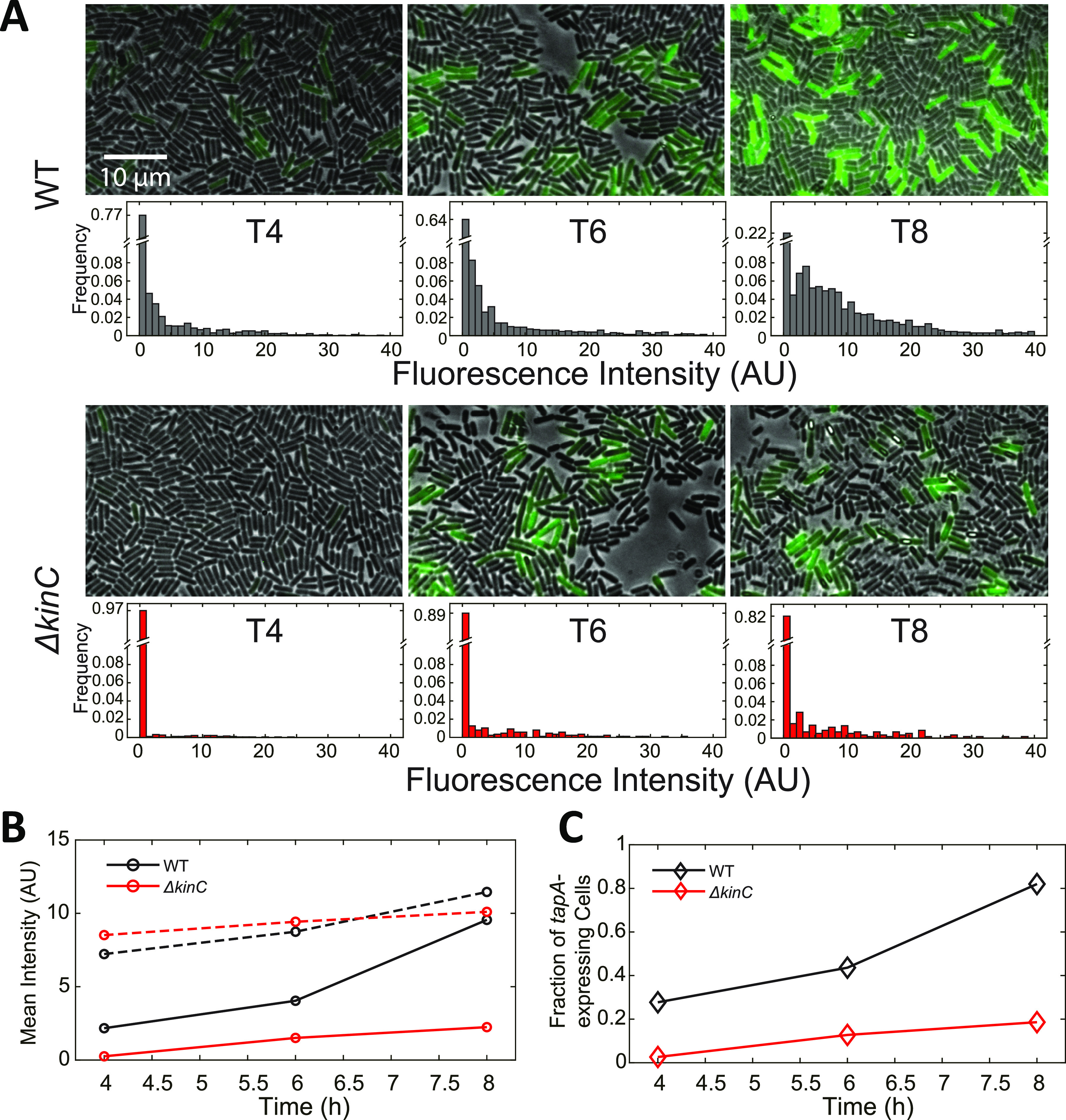
Fluorescence measurements for *PtapA* activity in individual cells. (A) Fluorescence-microscopic images of WT and Δ*kinC* cells harboring *PtapA-gfp* reporter at different times. The scale bar applies to all the images. The GFP channel is shown in green pseudocolor. Each of the strains was cultured in liquid MSgg. The distribution of mean fluorescence intensity of cells is plotted under each image. The value of the first bin was labeled on each histogram axis. (B) Mean fluorescence intensities of all cells (solid line) and *tapA*-expressing cells (dashed line) at T4, T6, and T8. (C) Fractions of *tapA*-expressing cells of WT and Δ*kinC* strains at T4, T6, and T8.

To experimentally test the model of the heterogeneity of *tapA* expression described in the above section, we performed single-cell measurements of *PtapA* activity using a fluorescent reporter (Fig. 5A; see Materials and Methods for details). The single-cell level distributions of *PtapA* activity were examined in the WT and Δ*kinC* strains after 4, 6, and 8 h of culture (defined as T4, T6, and T8, respectively) (Fig. 5A). We found that all of the distributions have a sharp peak near zero. These results indicate that the majority of cells do not express *tapA* except for the WT strain at T8. Excluding the peak, the fluorescence intensity values were widely distributed.

Further analysis indicates that the mean *PtapA* activity is mainly determined by the fraction of *tapA*-expressing cells instead of the *tapA* expression levels. To better understand the role of KinC in the regulation of *tapA* expression, we quantified green fluorescent protein (GFP) intensity values for “all cells” (including *tapA*-expressing and non-*tapA*-expressing cells) and for cells expressing with intensity significantly higher GFP values than the background as “*tapA*-expressing cells.” As Fig. 5B shows, the mean fluorescence intensity of the WT cells was higher than that of the Δ*kinC* cells, and these values increased with time, which is consistent with the *lacZ* reporter data (Fig. 1A). However, the mean fluorescence intensity of *tapA*-expressing cells was relatively constant both in the WT and Δ*kinC* strains at different times (Fig. 5B, dashed lines). In contrast, the dynamics of the fraction of *tapA*-expressing cells showed the same trend as the mean fluorescence intensity of all cells (compare solid lines in Fig. 5B and C). These results indicate that, as predicted by the model (Fig. 4), it is the fraction of *tapA*-expressing cells, rather than the *tapA* expression levels in individual cells, that determines the mean *tapA* expression level in a culture population.

### KinC has opposite effects on matrix production depending on KinA level.

**FIG 6:**
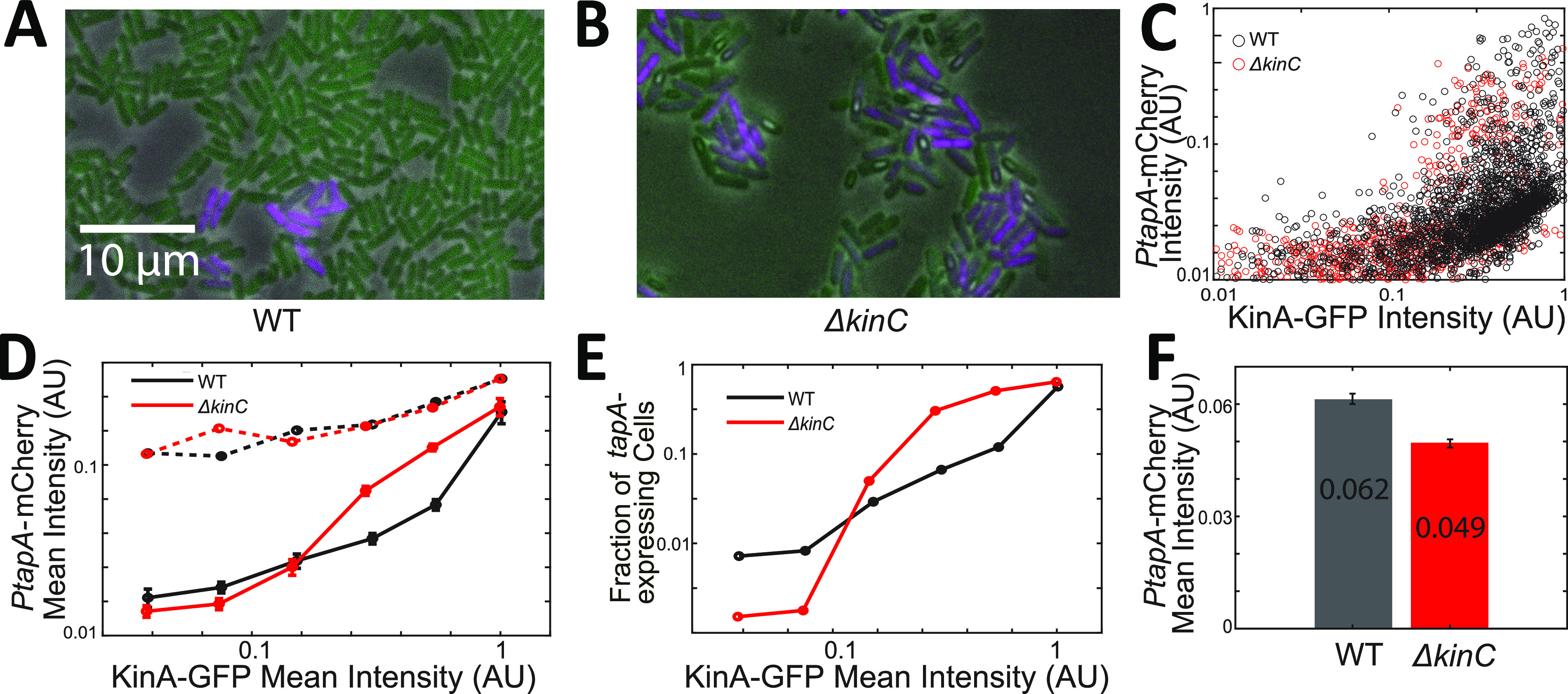
Fluorescence measurements for KinA levels and *PtapA* activity in individual cells. (A and B) Fluorescence-microscopic images of WT (A) and Δ*kinC* (B) cells harboring *PtapA-mCherry* and *kinA-GFP* taken after 6 to 10 h of culture. Each of the strains was cultured in liquid MSgg. The GFP and mCherry channels are shown in green and magenta pseudocolors, respectively. The scale bar applies to both A and B. (C) Scatterplot of the *PtapA*-mCherry and KinA-GFP intensities of all the WT and Δ*kinC* cells. Each circle represents one cell. (D) Mean *PtapA-mCherry* intensities of all cells (solid line) and *tapA*-expressing cells (dashed line) that have different KinA-GFP intensity. All the cells were categorized by their KinA-GFP intensity. The mean *PtapA*-mCherry and KinA-GFP intensities were then calculated for WT and Δ*kinC* strains, respectively. The error bars show the standard errors of the means for each category. (E) The fraction of *tapA*-expressing cells of WT and Δ*kinC* cells that have different KinA-GFP intensities. (F) Mean *PtapA-mCherry* intensities of all WT and Δ*kinC* cells regardless of their KinA level. The error bars show the standard errors of the means.

The results thus far show that experimental observations are consistent with the prediction that KinC mainly affects the fraction of *tapA*-expressing cells by modulating the heterogeneity of Spo0A∼P in single cells. However, these observations do not directly validate the hypothesis that KinC plays different roles at different levels of KinA (Fig. 2A). To directly test how the effect of KinC on Spo0A∼P depends on the concentration of KinA, we employed the strains in which single-cell levels of KinA (with a functional KinA-GFP fusion) and *PtapA* activity (with *PtapA*-mCherry) can be measured simultaneously (Fig. 6A and B; see Materials and Methods for details). These reporters were introduced in the WT and Δ*kinC* backgrounds. The fluorescent images were taken after 6 to 10 h of culture to get a wide distribution of KinA levels. The KinA-GFP and *PtapA*-mCherry intensities were quantified for all the cells in the field of view (*n *= 6,232 for WT and 5,524 for Δ*kinC*) (Fig. 6C). Then, we divided the cells into 6 bins based on the KinA-GFP intensity. For each bin, the mean *PtapA*-mCherry intensities of all cells (Fig. 6D, solid lines) and *tapA*-expressing cells (Fig. 6C, dashed line), as well as the fraction of *tapA*-expressing cells (Fig. 6E), were calculated. The mean *PtapA*-mCherry activities of all WT and Δ*kinC* cells are shown in Fig. 6F.

These results supported the hypothesis that KinC has opposite effects on *tapA* expression in the cells depending on KinA levels. In cells with low KinA levels, KinC increased the mean level of *tapA* expression by increasing the fraction of cells that activate it. The opposite trend was observed in the cells with high KinA: the presence of KinC negatively regulates *tapA* expression by decreasing the fraction of expressing cells (Fig. 6D and E). Notably, the negative effect of KinC on *tapA* expression was not observed at the population-average level (Fig. 6F), which is consistent with the β-galactosidase data (Fig. 1A). These results validate our predictions that the effect of KinC on Spo0A∼P depends on the level of KinA and that the negative effect of KinC is “concealed” by the heterogeneity of cellular KinA concentrations.

## DISCUSSION

In B. subtilis, both sporulation and biofilm matrix production genes are activated by a single master regulator, Spo0A, which is activated by phosphorylation through the phosphorelay network initiated via multiple kinases. Despite the similarity of their biochemical mechanisms, different kinases, including KinA and KinC, have distinct effects on downstream gene expression. Here, we unraveled the mechanism by which KinC positively regulates biofilm matrix production but negatively controls sporulation (Fig. 1). Using mathematical modeling, we showed that the dual roles of KinC could be explained with three key features of the phosphorelay network. (i) Depending on the level of KinA, KinC could act as the phosphate source or sink. (ii) Growth rate affects the cellular concentration of both KinA and KinC but in different manners. (iii) KinC increases the fraction of *tapA*-expressing cells in a culture population by reducing the heterogeneity of Spo0A∼P concentrations in individual cells. As discussed below, these three features explain how KinC differently regulates matrix production and sporulation.

### KinC acts as a phosphate source or sink depending on the concentration of KinA.

The experimental results indicate that after 5 h of culture, the expression from the Spo0A∼P-activated *spoIIG* promoter is higher in the Δ*kinC* strain than in the WT strain (Fig. 1B). Notably, while the negative effect of KinC on *tapA* expression is not observed at the population-average level (Fig. 1A and 6F), the single-cell-level data show that KinC negatively affects *PtapA* activity in the cells that have high levels of KinA (Fig. 6D). These observations suggests that KinC negatively affects Spo0A∼P concentrations at the late stage of starvation. Our mathematical model explains this by demonstrating that KinC can act as the phosphate sink, competing with Spo0A phosphorylation flux from KinA. The model is based on the fact that all the phosphotransfer reactions in the phosphorelay are reversible and different kinases are expected to have different kinetic parameters (19, 33, 34). Such a phosphate sink mechanism has also been observed in other bacterial multikinase networks controlling virulence and chemotaxis (44–46).

Bacterial histidine kinases are known to possess not only kinase but also phosphatase activities (47). Notably, another phosphorelay kinase, KinD, has been reported to repress sporulation by acting as a phosphatase that reduces Spo0A∼P (48). Thus, an alternative explanation for the negative effect of KinC on Spo0A∼P would be the existence of phosphatase activity of KinC toward Spo0A or Spo0F. Indeed, in our model, introducing the phosphatase activity of KinC would not qualitatively change the ability of KinC to act as the phosphate source or sink depending on the levels of KinA (Fig. S5). Thus, either phosphatase activity or reverse phosphotransfer activity can explain the distinct roles of KinC. However, the evidence from *in vitro* experiments suggests that KinC dephosphorylates Spo0F∼P through reverse phosphotransfer and reverse autophosphorylation reactions instead of directly hydrolyzing phosphate (20, 34). Indeed, reverse phosphotransfer from Spo0F∼P to KinA and reverse autophosphorylation of KinA have been observed both *in vitro* (34, 49) and *in vivo* (37, 38). Moreover, *in vitro* experiments showed that in the presence of ADP, KinC possesses kinetics similar to that of KinA for the dephosphorylation of Spo0F∼P, producing ATP (20), suggesting the shared mechanism of reverse phosphotransfer followed by reverse autophosphorylation reaction.

10.1128/mBio.01694-21.5 FIG S5 Introducing the phosphate activity of KinC into the model would not change the distinct roles of KinC at different KinA levels. (A) Predicted fraction of Spo0A∼P as a function of KinA and KinC concentrations. The black dashed lines are the contour lines of Spo0A∼P fraction. The green and blue dashed lines mark the KinA levels corresponding to the green and blue curves in panel B. (B) The predicted fraction of Spo0A∼P changes with KinC levels in different manners under different levels of KinA. The dashed lines show the Spo0A∼P fractions at intermediate KinA concentrations (0.1 μM, 0.4 μM, 0.8 μM, 1.2 μM, 1.6 μM, and 2 μM). Download FIG S5, PDF file, 0.5 MB. Copyright © 2022 Chen et al. 2022 Chen et al. https://creativecommons.org/licenses/by/4.0/ This content is distributed under the terms of the Creative Commons Attribution 4.0 International license.

Critically, our mathematical model shows that the direction of the phosphotransfer reaction between KinC and Spo0F is determined by the concentration of KinA (Fig. 2). Due to differences in the kinetic parameters of KinA and KinC, KinC always tends to maintain low Spo0A∼P concentrations, whereas KinA tends to maintain high Spo0A∼P concentrations. At low levels of KinA, KinC provides the source of phosphate and generates a low concentration of Spo0A∼P. When KinA levels are high, the resulting high flux of phosphate toward Spo0A in phosphorelay leads to high Spo0A∼P concentrations. Under these conditions, KinC pulls phosphate from Spo0F∼P via reverse phosphotransfer, competing with the phosphate flux toward Spo0F from KinA. Subsequent reverse autophosphorylation of KinC acts as a phosphate sink. These results provide a novel mechanism for bacteria to fine-tune the phosphorylation level of a transcription factor by balancing the dosages of different kinases. This mechanism may be employed in a wide range of multikinase systems.

### Growth rate affects the cellular concentrations of both KinA and KinC but in different manners.

Growth rate changes affect the cellular concentrations of proteins via their effects on DNA replication, dilution, cell volume, and transcription/translation rates (50). The global effect of growth rate is known to affect the behavior of multiple gene regulatory circuits (39, 51–53). Notably, the slowdown of growth leads to the accumulation of KinA, thereby causing the increase in Spo0A∼P concentrations and triggering sporulation (26).

The effect of growth rate on protein concentrations is determined by protein stability. Indeed, the slowdown of growth mainly affects cellular protein concentrations by decreasing the protein dilution rates (54). In our model, the effect of protein dilution (dilution rate [*k*_dil_], equal to the growth rate) is additive with protein degradation (nonspecific degradation rate [*k*_deg_]). As a result, the effective protein decay rate is represented as *k*_deg_ + *k*_dil_. The steady-state protein concentration is determined by the ratio of the production rate and this effective decay rate. Therefore, the effect of growth on protein concentration is dependent on the relative values of *k*_dil_ and *k*_deg_. For unstable proteins such as Sda (25), *k*_deg_ is much greater than *k*_dil_, so the changes of *k*_dil_ do not significantly change protein concentrations. On the other hand, for stable proteins such as KinA, *k*_deg_ is much lower than *k*_dil_, so the changes of growth will greatly affect the protein concentrations. Therefore, the model predicts that the relative concentrations of KinA and its stoichiometric inhibitor Sda are highly dependent on growth rate: when the growth rate is high, Sda concentration is higher than KinA concentration and the majority of KinA is kept in the inactive state in the KinA-Sda complex at a 1:1 stoichiometric ratio. However, when the growth rate decreases, KinA concentration exceeds Sda concentration (Fig. S2B and C), leading to a large increase in the concentration of free (unbound) KinA (Fig. 3A). This “molecular titration” mechanism (55), resulting from sequestration of a stable protein by an unstable partner, provides a simple mechanism for cells to ultrasensitively respond to the changes of growth rate. Such a mechanism is known to play an important role in the toxin-antitoxin systems in bacteria (53, 56–58). Since Sda has very low affinity of binding to KinC compared with KinA (23), most of the KinC molecules are present in a free and active form. Thus, KinC activity is not expected to be as sensitive to the changes of growth.

Differential sensitivity of KinA and KinC to growth rate allows our model to explain distinct effects of KinC on Spo0A∼P at different growth stages. We predicted that when the cell growth rate is high at early stages of starvation, KinA is inactive and KinC is predominantly responsible for Spo0A phosphorylation. In contrast, under slow-growth conditions at later stages of starvation, KinA becomes active and plays a major role in Spo0A phosphorylation. As a result, KinC switches its role from the phosphate source to the phosphate sink and thereby counteracts KinA activity. This source-to-sink switch effect allows KinC to weaken the growth rate dependence of Spo0A∼P by increasing its concentration at the early stages of starvation and decreasing it at the late stages of starvation. As discussed below, this effect allows KinC to reduce the cell-to-cell heterogeneity in the Spo0A∼P concentrations and thereby active matrix production in a larger fraction of cells.

Unfortunately, technical limitations did not allow us to directly demonstrate the differential roles of KinC in single cells with different growth rates as predicted by the model. Quantification of single-cell gene expression with time-lapse microscopy of a biofilm-producing strain on an agarose pad is very challenging. For example, in time-lapse microscopy imaging, a coverslip placed on top of the biofilm can interfere with its three-dimensional growth. Moreover, a high density of cells in the biofilm does not allow effective single-cell segmentation. Nevertheless, we indirectly confirmed the prediction by taking snapshots of single cells from the liquid culture with the dual reporters KinA-GFP and *PtapA*-mCherry. Our results demonstrated that the effect of KinC on *PtapA* activity depends on the level of KinA (Fig. 6). Given the fact that the KinA level is closely correlated with growth rate (26), our experimental results support our conclusion that the effect of KinC on Spo0A∼P depends on the growth rate.

### KinC increases the fraction of *tapA*-expressing cells by reducing the heterogeneity of Spo0A∼P concentrations.

Matrix production is known to be heterogeneous in B. subtilis cell communities (7). As shown in Fig. 5A, our results confirm that only a small fraction of the cells express matrix genes (14, 59, 60). Our results also show that the per-cell *PtapA* activity in the *tapA*-expressing population remains constant, while the fraction of *tapA*-expressing cells increases with time. Moreover, the deletion of *kinC* results in a lower fraction of *tapA*-expressing cells. These results indicate that the fraction of *tapA*-expressing cells in the population, instead of the *tapA* expression level in individual cells, is the major determinant for the population-level mean *tapA* expression level.

The heterogeneity of matrix production is affected by the noise in growth rate. The growth rates of individual cells in a B. subtilis population are known to be highly heterogeneous (26). Some recent studies showed that the noise in growth rate plays an important role in determining the heterogeneity of protein expression and the behavior of various gene regulatory networks (54, 61–63). Given that Spo0A∼P levels in individual cells are determined by the growth rate (26), the noise in growth rate plays an important role in regulating the heterogeneity of Spo0A∼P levels. The noise in the phosphorelay network may also affect the heterogeneity of Spo0A∼P levels (64, 65), but a previous study showed that the heterogeneity of sporulation could be explained by the noise in growth rate (26), suggesting that the noise in Spo0A∼P levels is largely determined by the noise in growth rate.

Critically, our model qualitatively explains how KinC increases the fraction of *tapA*-activated cells by changing the relationship between Spo0A∼P concentration and growth rate on a single-cell level (Fig. 4A). For the relatively fast-growing cells in the heterogeneous population, KinA is inactive, and thus, KinC contributes to increasing Spo0A∼P levels and thereby *tapA* expression. On the other hand, for the relatively slow-growing cells, KinA concentrations are high, so the Spo0A∼P levels are more than sufficient to saturate the activation of *tapA* transcription regardless of KinC but still not sufficient for the saturation of *spoIIG*. Therefore, the phosphate sink through KinC would reduce Spo0A∼P levels and reduce the expression of sporulation genes but not matrix production genes. As a result, the presence of KinC increases the fraction of *tapA*-expressing cells even though the mean Spo0A∼P level is reduced and sporulation is repressed.

Notably, the distribution of *tapA* expression levels (Fig. 5A) does not exactly match the model predictions (Fig. 4). The fractions of *tapA*-expressing cells are much lower than expected; even in the WT strain, most cells were not expressing *tapA* at T4 and T6 (Fig. 5A). These results suggest that there should be other sources of noise affecting the expression of *tapA* that is not captured by the model, for example, the noise in the downstream SinI-SinR-SlrR regulatory network. Matrix production is controlled by the bistable regulatory network consisting of SinI, SinR, and SlrR (14, 16). The stochastic fluctuations in the SinI-SinR-SlrR network largely determine the heterogeneous expression of *tapA* (66, 67). Thus, it is possible that the intrinsic noise in the SinI-SinR-SlrR regulatory network would affect the distribution of *tapA* expression level and reduce the fraction of *tapA*-expressing cells. Properly accounting for this intrinsic noise will be a subject of the further studies.

### Conclusions.

In summary, our results indicate a mechanistic role for KinC in controlling biofilm matrix production and sporulation gene expression. Despite acting as a phosphoryl-group sink and thereby negatively affecting mean Spo0A∼P level and sporulation under slow-growth conditions, KinC nevertheless always positively regulates matrix production by reducing the noise in Spo0A∼P among individual cells in a population. This noise reduction increases the fraction of cells with a Spo0A∼P concentration high enough to allow the expression of *tapA*. Therefore, our results reveal that considering gene expression stochasticity and population-level heterogeneity could be essential to even qualitatively understand the population mean response to genetic perturbations. Such effects can undoubtedly play a role in a wide range of other biological systems.

## MATERIALS AND METHODS

### Experimental methods. (i) Strains and plasmids.

The B. subtilis strains used in this work are isogenic derivatives of the undomesticated and competent DK1042 (68). DK1042 is a derivative of strain NCIB3610 (3) and also available as B. subtilis 3A38 at the *Bacillus* Genetic Stock Center (BGSC), Ohio State University, Columbus, OH, USA. All mutant strains of B. subtilis were constructed by transformation with either chromosomal DNA or plasmid DNA as described by Harwood and Cutting (69). The standard recombinant DNA techniques, including plasmid DNA construction and isolation using Escherichia coli DH5a, were performed as described by Sambrook and Russell (70). To generate *thrC*::*PtapA-gfp erm* (pMF719) and *amyE*::*PtapA-mCherry spc* (pMF1130) strains, a DNA fragment containing *PtapA* was prepared from pMF712 (*PtapA-lacZ*) (27) with EcoRI and HindIII digestion. PCR fragments of the *gfp* and *mCherry* coding sequences were prepared as described previously (36). The reporter gene fragments (*gfp* and *mCherry*) were digested with HindIII and BamHI. The resulting fragments (*PtapA-gfp* and *PtapA-mCherry*) were cloned into pDG1664 (71) digested with EcoRI and BamHI to generate pMF719. The fragments of *PtapA* and *mCherry* were cloned into pDG1730 digested with EcoRI and BamHI to generate pMF1130. The resulting plasmids were inserted by double-crossover recombination into the *thrC* or *amyE* locus of the B. subtilis chromosome. *Bacillus* knockout erythromycin (BKE) strains for *kinA*, *kinB*, *kinC*, *kinD*, and *sda* were acquired from the *Bacillus* Genetics Stock Center. Markerless deletion mutants for *kinA kinB*, *kinC*, and *kinD* were constructed using the Cre-lox system (72). A strain harboring Phy-*spank-kinA* was generated as described previously (42). The KinA-inducible strain was constructed by introducing the Phy-*spank-kinA* construct into the *PtapA-lacZ* reporter strain (42). The strain harboring *kinA-gfp* was generated as described previously (42). The strain harboring *PtapA-mCherry* and *kinA-gfp* was generated by introducing the *PtapA-mCherry* construct into the *kinA-gfp* strain. A *thrC*::*PspoIIG-lacZ* construct was generated by inserting an EcoRI and HindIII fragment containing the *PspoIIG* portion derived from pMF27 (*amyE*::*PspoIIG-lacZ*) (73) into pDG1663 digested by the same restriction enzymes (71). The strains and plasmids used in this study are listed in Table S2.

10.1128/mBio.01694-21.8 TABLE S2 Strains and plasmids used in this work. Download Table S2, PDF file, 0.1 MB. Copyright © 2022 Chen et al. 2022 Chen et al. https://creativecommons.org/licenses/by/4.0/ This content is distributed under the terms of the Creative Commons Attribution 4.0 International license.

### (ii) Media and culture conditions.

Lysogeny broth (LB) medium (70) was used for routine growth of E. coli and B. subtilis. Difco sporulation medium (DSM) was used for sporulation of B. subtilis (69). Minimal salts glycerol glutamate (MSgg) was used for biofilm formation and sporulation of B. subtilis (3). Cells were cultured with shaking (150 rpm) overnight in LB (5 mL) at 28°C. The overnight culture was transferred to fresh LB (10 mL) to an optical density at 600 nm (OD_600_) of 0.05. The fresh culture was incubated at 37^∘^C with shaking (150 rpm) to the mid-log phase (OD_600_ ≈ 0.5) to synchronize cell growth. Then, the fresh culture was transferred to MSgg (20 mL) to an OD_600_ of 0.05 and incubated in a culture flask at 37°C with shaking (150 rpm). Culture samples were collected at the indicated time points and assayed for specific β-galactosidase activity or processed for microscopy. Cell growth in liquid medium was measured using a spectrophotometer by reading the OD_600_. When solid agar medium was made, 1.5% (wt/vol) agar was included. Strains harboring reporter genes at the nonessential *thr* locus were supplemented with 1 mg mL^−1^ of l-threonine in the MSgg medium. IPTG (10 μM) was added to the MSgg cultures as needed after an OD_600_ of 0.2 was reached. Antibiotics were used for the selection of transformants at the following concentrations: 10 μg mL^−1^ of tetracycline, 100 μg mL^−1^ of spectinomycin, 20 μg mL^−1^ of kanamycin, 5 μg mL^−1^ chloramphenicol, and 1 μg mL^−1^ of erythromycin.

### Assays and analysis. (i) β-Galactosidase assay.

B. subtilis undomesticated strains were grown in liquid medium as described in “Media and culture conditions” above. Samples were collected at indicated time points, and β-galactosidase assays were performed as described elsewhere (74). In brief, an aliquot of cells were suspended in 0.5 mL Z buffer (40 mM NaH_2_PO_4_·H_2_O, 60 mM Na_2_HPO_4_·7H_2_O, 1 mM MgSO_4_·7H_2_O, 10 mM KCl, and 38 mM 2-mercaptoethanol). Lysozyme was added to each sample to a final concentration of 0.2 mg mL^−1^ and incubated at 30°C for 15 min. The reaction was started with 200 μL of substrate *ortho*-nitrophenyl-β-galactoside (ONPG; 4 mg mL^−1^ of Z buffer). The reaction samples were incubated at 30^∘^C until sufficient yellow color had developed and stopped with 250 μL 1 M Na_2_CO_3_. After centrifuging the samples for 20 min at 15,000 × *g*, the OD_420_ and OD_550_ of the supernatant of the reaction mixture were measured. The absorbance at 550 can correct for light scattering from the residual cell debris after centrifugation. The relationship between OD_420_ and OD_550_ is described by the following equation: light scattering at OD_420_ = 1.75 × OD_550_. The β-galactosidase specific activity was calculated according to the formula 1,000 × {[OD_420_ − (1.75 × OD_550_)]/(*t* × *V* × OD_600_)}, where *t* is the reaction time in minutes, *V* is the volume of culture used in milliliters, and OD_600_ is the density of the culture. As Fig. S3C shows, the growth dynamics in the Δ*kinC* and Δ*kinA* strains were similar to that in the wild-type strain (given by OD_600_). That means OD_600_ is not affected by the matrix production, so it is safe to normalize β-galactosidase activity to OD_600_. The reading at OD_420_ is a combination of absorbance by *o*-nitrophenol (a yellow end product) and light scattering by cell debris. The mean activities of at least three independent experiments are shown, with standard deviations.

### (ii) Sporulation assay.

Cells were cultured as described in “Media and culture conditions” above. The number of spores per milliliter was determined as the total number of CFU on DSM agar plates after heating the serially diluted culture samples to 80°C for 10 min. The total number of viable cells was measured as the number of CFU in serially diluted culture samples without heat treatment. These numbers ranged around 5 × 10^8^ per mL for all strains. The mean and standard deviation for each medium from at least four independent experiments are shown.

### Microscopy analysis.

For preparation of cell samples for microscopy, a chamber was prepared by attaching Gene Frame (Thermo Scientific, AB-0577; 65 μL, 1.5 by 1.6 cm) to a slide glass (75) and filled with molten MSgg medium containing 1% (wt/vol) agarose (ISC Bioexpress, E-3119-500). Two microliters of cells grown in liquid MSgg medium was applied to the solid MSgg agarose in the Gene Frame chamber and covered by a cover glass. The resulting cell samples collected at the specified times were then immediately examined using a fluorescence microscope (Olympus, model BX61) with an Olympus UPlanFL N 100× microscope objective. GFP and mCherry fluorescence were visualized using Chroma 41017 and Olympus U-MWG2 filter sets, respectively. Typical exposure times were 200 ms. The microscope system control was performed using SlideBook image analysis software (Intelligent Imaging Innovations, Inc.). At least five different fields of view were taken at each time point from three biological replicates. Representative images are shown. GFP and mCherry channels are shown in green and magenta pseudocolors, respectively.

### Computational modeling methods. (i) Modeling the phosphorelay network.

To explain the effect of KinA and KinC on the regulation of Spo0A, we modified and extended a previous mathematical model of the phosphorelay network (26, 31). This model uses ordinary differential equations to describe the production/degradation of the phosphorelay components and the phosphotransfer reactions between them (Fig. 2A). The model also includes the autophosphorylation/reverse autophosphorylation of KinA and KinC and the dephosphorylation of Spo0A catalyzed by Spo0E. Notably, this model includes the inhibition of KinA by Sda. Sda is known to be a specific inhibitor of KinA (23), and our result shows that Sda is essential for the inhibition of KinA at early times (Fig. S2). Thus, we introduced Sda into the model. For each reaction, we explicitly included the intermediate complexes between the enzyme and substrate, and all the posttranslational reactions were modeled by mass action kinetics. The posttranslational reactions and corresponding kinetic parameters are shown in Table S1. The expression rates of all the species and corresponding parameters are also shown in Table S1. Corresponding differential equations are shown in Table S3.

10.1128/mBio.01694-21.7 TABLE S1 Parameters used in the mathematical models. Download Table S1, PDF file, 0.04 MB. Copyright © 2022 Chen et al. 2022 Chen et al. https://creativecommons.org/licenses/by/4.0/ This content is distributed under the terms of the Creative Commons Attribution 4.0 International license.

10.1128/mBio.01694-21.9 TABLE S3 The differential equations of the phosphorelay model. Download Table S3, PDF file, 0.08 MB. Copyright © 2022 Chen et al. 2022 Chen et al. https://creativecommons.org/licenses/by/4.0/ This content is distributed under the terms of the Creative Commons Attribution 4.0 International license.

The kinetic parameters of the phosphotransfer reactions between KinA, Spo0B, Spo0F, and Spo0A are the same as in reference 26. For simplicity, we assigned constant expression rates (v*_p_*) to KinA, KinC, Spo0F, Spo0E, and Spo0B. The expression of KinA and Spo0F are known to be regulated by Spo0A∼P directly and indirectly (via ςH) (76, 77). However, it is reported that the amounts of Spo0F and σ^H^ are not rate limiting for the accumulation of Spo0A∼P (64). Thus, in this model, we did not consider the feedback regulation of KinA and Spo0F expression. The disassociation rate and catalytic rate of KinC were estimated as described in reference 33.

To test the robustness of the model, we simulated our model with fluctuated parameters. All of the parameters of the phosphorelay model, including *k_a_*_1_, *k_a_*_2_, *k_c_*_1_, *k_c_*_2_, *k_a_*_3_, *k_a_*_4_, *k_c_*_3_, *k_c_*_4_, *k*_1_, *k*_2_, *k*_3_, *k*_4_, *k*_5_, *k*_6_, *k_i_*, *k_b_*, v*_f_*, v*_b_*, and v*_e_*, were sampled from a uniform distribution on the interval [*p_i_*/3, 3 *p_i_*], where *p_i_* is the original value of the parameter. One thousand different combinations of parameter values were tested. The results show that our conclusion that KinC positively affects Spo0A∼P at low levels of KinA and negatively affects Spo0A∼P at high levels of KinA holds for over 95% of cases (Fig. S6), so the conclusion is robust against the fluctuations of parameters.

To account for the alternative hypothesis that KinC has phosphate activity (Fig. S5), we modified the model. In this alternative model, the phosphate activity of KinC is described by the following equation:

(1) $$\text{KinC}\;\cdot \;\text{Spo0F}∼\text{P}\overset{k_{\mathrm{pc}}}{\to }\text{KinC}+\text{Spo0F}$$

Here, *k_pc_* was set to 20 h^−1^. Meanwhile, to eliminate the effect of reverse-phosphotransfer reaction from Spo0F to KinC, the other parameters of KinC were modified so they are the same as for KinA (*k_c_*_3_ = *k_a_*_3_; *k_c_*_4_ = *k_a_*_1_).

### (ii) Modeling the effect of growth rate on the protein concentration.

We assumed that the accumulation of KinA and KinC as cellular growth rate decreases drives the increase of Spo0A∼P levels. Thus, we explicitly modeled the global effect of growth rate on the cellular concentration of all the proteins. Following reference 31, the production rates of the proteins in the model were given by

(2) $$\text{v}=n{\text{v}}_{p}F(\mu )$$

Here, v*_p_* is the growth-rate independent gene expression rate, and *n* is the copy number of the gene. *F*(μ) is a phenomenological function describing how the cell volume changes with the growth rate that is phenomenologically written as:

(3) $$F(\mu )=2^{k(a-\mu )}$$

Following reference 78, we set *a *as 1.11 h^−1^ and *k *as 0.95 h.

Under our experimental conditions, the growth rate is relatively low, so the multifork replication is not considered. For simplicity, we assume that the replication starts right after cell division. Thus, following reference 50, the average copy number of a gene was estimated as

(4) $$n=2^{1-\tau_{c}p/\tau_{\text{cyc}}}$$

Here, τ_cyc_ is ln2/μ, which represents the length of the cell cycle, and τ*_c_* is the length of the C period. Following reference 78, τ*_c_* can be phenomenologically related to the growth rate (μ) as follows: τ*_c_* = 0.78 + 0.15/μ. *p* represents the position of the gene, i.e., the normalized distance between the gene and the replication origin. A value of 0 for *p* corresponds to the replication origin, and a value of 1 corresponds to the replication terminus. The positions of the genes in this model are shown in Table S1 based on data taken from the KEGG database.

In addition to affecting protein expression, growth rate also affects the dilution rate of proteins. According to Sekar and Hageman (79), we assumed that all the proteins except for Sda in the model are relatively stable, with the nonspecific degradation rate (*k*_deg_) fixed at 0.2 h^−1^. Sda is known to be subject to rapid degradation *in vivo*, so a larger degradation was assumed: *k*_deg_ for Sda was set to 9 h^−1^ (25). Then, the combined protein decay flux is given by

(5) $${\text{v}}_{d}=k_{d}\;\cdot \;c$$

where *c* is the concentration of the species and *k_d_* is given by the sum of degradation and dilution rate constants:

(6) $$k_{d}=k_{\text{deg}}+\mu$$

### (iii) Modeling promoter activities.

To reproduce the dynamics of *PspoIIG* and *PtapA* activities (Fig. 3C and D), we assumed that the activity of *PspoIIG* and *PtapA* are both determined by Spo0A∼P level with the Hill function:

(7) $$\mathrm{PtapA}\;\text{activity}\propto \frac{{\left[\text{Spo0A}∼\text{P}\right]}^{n_{t}}}{{\left[\text{Spo0A}∼\text{P}\right]}^{n_{t}}+K_{t}^{n_{t}}}$$

(8) $$\mathrm{PspoIIG}\;\text{activity}\propto \frac{{\left[\text{Spo0A}∼\text{P}\right]}^{n_{g}}}{{\left[\text{Spo0A}∼\text{P}\right]}^{n_{g}}+K_{g}^{n_{g}}}$$

As an alternative hypothesis (Fig. S4A), we introduced the repression of *tapA* expression by high Spo0A∼P levels. In that case, the activity of *PtapA* promoter is given by:

(9) $$\mathrm{PtapA}\;\text{activity}\propto \frac{{\left[\text{Spo0A}∼\text{P}\right]}^{n_{t1}}}{{\left[\text{Spo0A}∼\text{P}\right]}^{n_{t1}}+K_{t1}^{n_{t1}}}\frac{K_{t2}^{n_{t2}}}{{\left[\text{Spo0A}∼\text{P}\right]}^{n_{t2}}+K_{t2}^{n_{t2}}}$$

To determine the unknown parameters, including the Hill coefficient and half-maximal concentration of Spo0A∼P for *PtapA* and *PspoIIG* activity, we fitted our model to the experimental data. The model was simulated to get the steady-state values of *PspoIIG* and *PtapA* activities at different times. Then, the unknown parameters were optimized to minimize the total mean-square error between the simulated data and the experimental data using the particleswarm function of MATLAB. The fitted parameters are shown in Table S1. The alternative hypothesis that *tapA* was repressed by high Spo0A∼P levels was fitted independently. The result was plotted in Fig. S4A.

### (iv) Modeling the growth dynamics and heterogeneity.

We used a Moser-type model (80) to describe the growth dynamics. Following reference 78, we assumed that the cell death or sporulation would release some of the nutrients back to the environment. The following differential equations would describe our model:

(10) $$\frac{\mathrm{dC}}{\mathrm{dt}}=C\left(k_{g}\frac{N^{h_{1}}}{N^{h_{1}}+K_{1}^{h_{1}}}-k_{d}\frac{K_{2}^{h_{2}}}{N^{h_{2}}+K_{2}^{h_{2}}}\right)$$

(11) $$\frac{\mathrm{dN}}{\mathrm{dt}}=-\text{γ}C\left(k_{g}\frac{N^{h_{1}}}{N^{h_{1}}+K_{1}^{h_{1}}}-\text{ψ}k_{d}\frac{K_{2}^{h_{2}}}{N^{h_{2}}+K_{2}^{h_{2}}}\right)$$

Here, *N* denotes the nutrient level and *C* denotes the cell number. *k_g_* and *k_d_* represent the maximum growth rate and death/sporulation rate, respectively. *K*_1_ and *K*_2_ are the half-maximum nutrient levels of cell growth and death/sporulation, and *h*_1_ and *h*_2_ are the Hill coefficients. γ is the yield coefficient specifying the conversion between the nutrients and cell densities, and ψ is the fraction (0 < ψ < 1) of the nutrient released by cell death/sporulation. The initial value of *N*, *N*_0_, was normalized to 1, and then *K*_1_ and *K*_2_ were fitted. To be consistent with the experiment, we used the OD value to represent the cell density, and *C*_0_ was set to 0.1. The model was fitted to the growth curve (Fig. S3A) using the fmincon function of MATLAB. Note that the growth curves of different strains were similar (Fig. S3C), so we used the same growth dynamics model for all the strains. The fitted parameters are shown in Table S1.

To examine the effect of growth rate in the heterogeneity of *tapA* expression, following (43), we assume that the distribution of generation times (τ_cyc_) of B. subtilis cells could be approximated to a normal distribution. In the model, the generation times of cells were sampled from a normal distribution with a coefficient of variation (CV) of 0.25, and the mean generation time is calculated based on the mean growth rate determined by the time via the growth dynamics model. The growth rate is calculated as ln2/τ_cyc_. To avoid unrealistically high growth rates, we discarded the generation times below 0.2 h.

### (v) Simulation of the model.

To illustrate how Spo0A∼P levels regulated by KinA and KinC (Fig. 2), we modified the model of the phosphorelay network: the production rate of Spo0A was fixed at 1 μM h^−1^, and the growth rate was fixed at 0.5 h^−1^. The production rates of KinA and KinC were varied to get different concentrations of active KinA and KinC. The steady-state concentrations of Spo0A and Spo0A∼P were calculated, and the fraction of Spo0A∼P {i.e., [Spo0A∼P]/([Spo0A]+[Spo0A∼P]+ [Spo0E·Spo0A∼P]+[Spo0B·Spo0A∼P])} is plotted in Fig. 2B and C.

The model of the phosphorelay network was simulated at different growth rates to get the steady-state values for different species. The MATLAB function ode15s was used to solve the differential equations. Numerical simulations suggest that the system is monostable and the initial conditions would not affect the steady-state values. Using these models, we reproduced the dynamics of the concentration of different species (Fig. 3A and B; Fig. S2B and C), and the *PtapA* and *PspoIIG* activities (Fig. 3C and D; Fig. S3A) were then calculated.

To calculate the distribution of Spo0A∼P levels, we sampled 1,000 generation times from the distribution described in the section above. The distribution of the growth rate was then calculated and plotted in Fig. S3B. For each growth rate, the Spo0A∼P concentration was calculated. the resulting distribution of Spo0A∼P concentration was plotted in Fig. 4C. Then the distribution of *PtapA* (Fig. 4D) and *PspoIIG* activities (Fig. 4E) were calculated based on the distribution of Spo0A∼P concentration.

### Quantification of fluorescence images.

The cells were segmented based on the phase images using Oufti (81) and custom codes. The pixelwise mean fluorescence intensity was calculated for each segmented cell. For each image, the pixelwise mean fluorescence intensity of the no-cell area was calculated as background. The background was subtracted from the fluorescence intensity of cells. The pixel-wise standard error of the background, σ*_p_*, was calculated for each image. We assumed that background noise follows normal distribution, and the background noise is pixelwise independent. For a cell containing *n* pixels, if its fluorescence intensity follows the same distribution with the background, then the standard error of the mean fluorescence intensity (σ) should be σ*_p_*/*N*^1/2^. The intensity values higher than 3σ were considered to be significantly higher than the background, and cells with these values were designated TapA-expressing cells.

### Data and code availability.

The data and code used in this study can be found at https://doi.org/10.5281/zenodo.5701607.

## References

[1] Storz G, Hengge R. 2011. Bacterial stress responses, 2nd ed. ASM Press, Washington, DC.

[2] Kearns DB, Chu F, Branda SS, Kolter R, Losick R. 2005. A master regulator for biofilm formation by Bacillus subtilis. Mol Microbiol 55:739–749. doi:10.1111/j.1365-2958.2004.04440.x. 15661000

[3] Branda SS, Gonzalez-Pastor JE, Ben-Yehuda S, Losick R, Kolter R. 2001. Fruiting body formation by Bacillus subtilis. Proc Natl Acad Sci USA 98:11621–11626. doi:10.1073/pnas.191384198. 11572999 PMC58779

[4] Flemming HC, Wingender J, Szewzyk U, Steinberg P, Rice SA, Kjelleberg S. 2016. Biofilms: an emergent form of bacterial life. Nat Rev Microbiol 14:563–575. doi:10.1038/nrmicro.2016.94. 27510863

[5] Riley EP, Schwarz C, Derman AI, Lopez-Garrido J. 2020. Milestones in Bacillus subtilis sporulation research. Microb Cell 8:1–16. doi:10.15698/mic2021.01.739. 33490228 PMC7780723

[6] van Gestel J, Vlamakis H, Kolter R. 2015. From cell differentiation to cell collectives: Bacillus subtilis uses division of labor to migrate. PLoS Biol 13:e1002141. doi:10.1371/journal.pbio.1002141. 25894589 PMC4403855

[7] Vlamakis H, Chai Y, Beauregard P, Losick R, Kolter R. 2013. Sticking together: building a biofilm the Bacillus subtilis way. Nat Rev Microbiol 11:157–168. doi:10.1038/nrmicro2960. 23353768 PMC3936787

[8] Hoch JA. 1993. Regulation of the phosphorelay and the initiation of sporulation in Bacillus subtilis. Annu Rev Microbiol 47:441–465. doi:10.1146/annurev.mi.47.100193.002301. 8257105

[9] Hamon MA, Lazazzera BA. 2001. The sporulation transcription factor Spo0A is required for biofilm development in Bacillus subtilis. Mol Microbiol 42:1199–1209. doi:10.1046/j.1365-2958.2001.02709.x. 11886552

[10] Fujita M, Gonz Ález-Pastor JE, Losick R. 2005. High- and low-threshold genes in the Spo0A regulon of Bacillus subtilis. J Bacteriol 187:1357–1368. doi:10.1128/JB.187.4.1357-1368.2005. 15687200 PMC545642

[11] Molle V, Fujita M, Jensen ST, Eichenberger P, González-Pastor JE, Liu JS, Losick R. 2003. The Spo0A regulon of Bacillus subtilis. Mol Microbiol 50:1683–1701. doi:10.1046/j.1365-2958.2003.03818.x. 14651647

[12] Narula J, Devi SN, Fujita M, Igoshin OA. 2012. Ultrasensitivity of the Bacillus subtilis sporulation decision. Proc Natl Acad Sci USA 109:E3513–E3522. doi:10.1073/pnas.1213974109. 23169620 PMC3528541

[13] Eswaramoorthy P, Dinh J, Duan D, Igoshin OA, Fujita M. 2010. Single-cell measurement of the levels and distributions of the phosphorelay components in a population of sporulating Bacillus subtilis cells. Microbiology (Reading) 156:2294–2304. doi:10.1099/mic.0.038497-0. 20413551

[14] Chai Y, Chu F, Kolter R, Losick R. 2007. Bistability and biofilm formation in Bacillus subtilis. Mol Microbiology 67:254–263. doi:10.1111/j.1365-2958.2007.06040.x. PMC2430929 18047568

[15] Bai U, Mandic-Mulec I, Smith I. 1993. SinI modulates the activity of SinR, a developmental switch protein of Bacillus subtilis, by protein-protein interaction. Genes Dev 7:139–148. doi:10.1101/gad.7.1.139. 8422983

[16] Chai Y, Norman T, Kolter R, Losick R. 2010. An epigenetic switch governing daughter cell separation in Bacillus subtilis. Genes Dev 24:754–765. doi:10.1101/gad.1915010. 20351052 PMC2854391

[17] Kobayashi K. 2008. SlrR/SlrA controls the initiation of biofilm formation in Bacillus subtilis. Mol Microbiol 69:1399–1410. doi:10.1111/j.1365-2958.2008.06369.x. 18647168

[18] Chu F, Kearns DB, Branda SS, Kolter R, Losick R. 2006. Targets of the master regulator of biofilm formation in Bacillus subtilis. Mol Microbiol 59:1216–1228. doi:10.1111/j.1365-2958.2005.05019.x. 16430695

[19] Burbulys D, Trach KA, Hoch JA. 1991. Initiation of sporulation in B. subtilis is controlled by a multicomponent phosphorelay. Cell 64:545–552. doi:10.1016/0092-8674(91)90238-T. 1846779

[20] Jiang M, Shao W, Perego M, Hoch JA. 2000. Multiple histidine kinases regulate entry into stationary phase and sporulation in Bacillus subtilis. Mol Microbiol 38:535–542. doi:10.1046/j.1365-2958.2000.02148.x. 11069677

[21] LeDeaux JR, Yu N, Grossman AD. 1995. Different roles for KinA, KinB, and KinC in the initiation of sporulation in Bacillus subtilis. J Bacteriol 177:861–863. doi:10.1128/jb.177.3.861-863.1995. 7836330 PMC176674

[22] Ohlsen KL, Grimsley JK, Hoch JA. 1994. Deactivation of the sporulation transcription factor Spo0A by the Spo0E protein phosphatase. Proc National Acad Sci 91:1756–1760. doi:10.1073/pnas.91.5.1756. PMC43242 8127878

[23] Burkholder WF, Kurtser I, Grossman AD. 2001. Replication initiation proteins regulate a developmental checkpoint in Bacillus subtilis. Cell 104:269–279. doi:10.1016/s0092-8674(01)00211-2. 11207367

[24] Rowland SL, Burkholder WF, Cunningham KA, Maciejewski MW, Grossman AD, King GF. 2004. Structure and mechanism of action of Sda, an inhibitor of the histidine kinases that regulate initiation of sporulation in Bacillus subtilis. Mol Cell 13:689–701. doi:10.1016/S1097-2765(04)00084-X. 15023339

[25] Ruvolo MV, Mach KE, Burkholder WF. 2006. Proteolysis of the replication checkpoint protein Sda is necessary for the efficient initiation of sporulation after transient replication stress in Bacillus subtilis. Mol Microbiol 60:1490–1508. doi:10.1111/j.1365-2958.2006.05167.x. 16796683

[26] Narula J, Kuchina A, Zhang F, Fujita M, Süel GM, Igoshin OA. 2016. Slowdown of growth controls cellular differentiation. Mol Syst Biol 12:871. doi:10.15252/msb.20156691. 27216630 PMC5289222

[27] Devi SN, Vishnoi M, Kiehler B, Haggett L, Fujita M. 2015. In vivo functional characterization of the transmembrane histidine kinase KinC in Bacillus subtilis. Microbiology (Reading) 161:1092–1104. doi:10.1099/mic.0.000054. 25701730

[28] Romero D, Vlamakis H, Losick R, Kolter R. 2014. Functional analysis of the accessory protein TapA in Bacillus subtilis amyloid fiber assembly. J Bacteriol 196:1505–1513. doi:10.1128/JB.01363-13. 24488317 PMC3993358

[29] Jonas RM, Weaver EA, Kenney TJ, Moran CP, Haldenwang WG. 1988. The Bacillus subtilis spoIIG operon encodes both sigma E and a gene necessary for sigma E activation. J Bacteriol 170:507–511. doi:10.1128/jb.170.2.507-511.1988. 2448286 PMC210682

[30] Fujita M, Losick R. 2003. The master regulator for entry into sporulation in Bacillus subtilis becomes a cell-specific transcription factor after asymmetric division. Genes Dev 17:1166–1174. doi:10.1101/gad.1078303. 12730135 PMC196045

[31] Narula J, Kuchina A, Lee DYD, Fujita M, Süel GM, Igoshin OA. 2015. Chromosomal arrangement of phosphorelay genes couples sporulation and DNA replication. Cell 162:328–337. doi:10.1016/j.cell.2015.06.012. 26165942 PMC4506695

[32] Vishnoi M, Narula J, Devi SN, Dao HA, Igoshin OA, Fujita M. 2013. Triggering sporulation in Bacillus subtilis with artificial two-component systems reveals the importance of proper Spo0A activation dynamics. Mol Microbiol 90:181–194. doi:10.1111/mmi.12357. 23927765

[33] Jiang M, Tzeng YL, Feher VA, Perego M, Hoch JA. 1999. Alanine mutants of the Spo0F response regulator modifying specificity for sensor kinases in sporulation initiation. Mol Microbiol 33:389–395. doi:10.1046/j.1365-2958.1999.01481.x. 10411754

[34] Grimshaw CE, Huang S, Hanstein CG, Strauch MA, Burbulys D, Wang L, Hoch JA, Whiteley JM. 1998. Synergistic kinetic interactions between components of the phosphorelay controlling sporulation in Bacillus subtilis. Biochemistry 37:1365–1375. doi:10.1021/bi971917m. 9477965

[35] Eswaramoorthy P, Dravis A, Devi SN, Vishnoi M, Dao HA, Fujita M. 2011. Expression level of a chimeric kinase governs entry into sporulation in Bacillus subtilis. J Bacteriol 193:6113–6122. doi:10.1128/JB.05920-11. 21926229 PMC3209216

[36] Eswaramoorthy P, Duan D, Dinh J, Dravis A, Devi SN, Fujita M. 2010. The threshold level of the sensor histidine kinase KinA governs entry into sporulation in Bacillus s. J Bacteriol 192:3870–3882. doi:10.1128/JB.00466-10. 20511506 PMC2916370

[37] Eswaramoorthy P, Guo T, Fujita M. 2009. In vivo domain-based functional analysis of the major sporulation sensor kinase, KinA, in Bacillus subtilis. J Bacteriol 191:5358–5368. doi:10.1128/JB.00503-09. 19561131 PMC2725609

[38] Kiehler B, Haggett L, Fujita M. 2017. The PAS domains of the major sporulation kinase in Bacillus subtilis play a role in tetramer formation that is essential for the autokinase activity. Microbiologyopen 6:e00481. doi:10.1002/mbo3.481. PMC5552956 28449380

[39] Klumpp S, Hwa T. 2014. Bacterial growth: global effects on gene expression, growth feedback and proteome partition. Curr Opin Biotechnol 28:96–102. doi:10.1016/j.copbio.2014.01.001. 24495512 PMC4111964

[40] Veening JW, Murray H, Errington J. 2009. A mechanism for cell cycle regulation of sporulation initiation in Bacillus subtilis. Genes Dev 23:1959–1970. doi:10.1101/gad.528209. 19684115 PMC2725940

[41] Chai Y, Norman T, Kolter R, Losick R. 2011. Evidence that metabolism and chromosome copy number control mutually exclusive cell fates in Bacillus subtilis. EMBO J 30:1402–1413. doi:10.1038/emboj.2011.36. 21326214 PMC3094124

[42] Fujita M, Losick R. 2005. Evidence that entry into sporulation in Bacillus subtilis is governed by a gradual increase in the level and activity of the master regulator Spo0A. Genes Dev 19:2236–2244. doi:10.1101/gad.1335705. 16166384 PMC1221893

[43] van Heerden JH, Kempe H, Doerr A, Maarleveld T, Nordholt N, Bruggeman FJ. 2017. Statistics and simulation of growth of single bacterial cells: illustrations with B. subtilis and E. coli. Sci Rep 7:16094. doi:10.1038/s41598-017-15895-4. 29170466 PMC5700928

[44] Francis VI, Waters EM, Finton-James SE, Gori A, Kadioglu A, Brown AR, Porter SL. 2018. Multiple communication mechanisms between sensor kinases are crucial for virulence in Pseudomonas aeruginosa. Nat Commun 9:2219. doi:10.1038/s41467-018-04640-8. 29880803 PMC5992135

[45] Amin M, Kothamachu VB, Feliu E, Scharf BE, Porter SL, Soyer OS. 2014. Phosphate sink containing two-component signaling systems as tunable threshold devices. PLoS Comput Biol 10:e1003890. doi:10.1371/journal.pcbi.1003890. 25357192 PMC4214558

[46] Tindall MJ, Porter SL, Maini PK, Armitage JP. 2010. Modeling chemotaxis reveals the role of reversed phosphotransfer and a bi-functional kinase-phosphatase. PLoS Comput Biol 6:e1000896. doi:10.1371/journal.pcbi.1000896. 20808885 PMC2924250

[47] Salazar ME, Laub MT. 2015. Temporal and evolutionary dynamics of two-component signaling pathways. Curr Opin Microbiol 24:7–14. doi:10.1016/j.mib.2014.12.003. 25589045 PMC4380680

[48] Aguilar C, Vlamakis H, Guzman A, Losick R, Kolter R. 2010. KinD is a checkpoint protein linking spore formation to extracellular-matrix production in Bacillus subtilis biofilms. mBio 1:e00035-10. doi:10.1128/mBio.00035-10. 20689749 PMC2912670

[49] Wang L, Fabret C, Kanamaru K, Stephenson K, Dartois V, Perego M, Hoch JA. 2001. Dissection of the functional and structural domains of phosphorelay histidine kinase A of Bacillus subtilis. J Bacteriol 183:2795–2802. doi:10.1128/JB.183.9.2795-2802.2001. 11292798 PMC99495

[50] Klumpp S, Zhang Z, Hwa T. 2009. Growth rate-dependent global effects on gene expression in bacteria. Cell 139:1366–1375. doi:10.1016/j.cell.2009.12.001. 20064380 PMC2818994

[51] Jaruszewicz-Błońska J, Lipniacki T. 2017. Genetic toggle switch controlled by bacterial growth rate. BMC Syst Biol 11:117. doi:10.1186/s12918-017-0483-4. 29197392 PMC5712128

[52] Liao C, Blanchard AE, Lu T. 2017. An integrative circuit–host modelling framework for predicting synthetic gene network behaviours. Nat Microbiol 2:1658–1666. doi:10.1038/s41564-017-0022-5. 28947816

[53] Feng J, Kessler DA, Ben-Jacob E, Levine H. 2014. Growth feedback as a basis for persister bistability. Proc Natl Acad Sci USA 111:544–549. doi:10.1073/pnas.1320396110. 24344277 PMC3890803

[54] Nordholt N, van Heerden J, Kort R, Bruggeman FJ. 2017. Effects of growth rate and promoter activity on single-cell protein expression. Sci Rep 7:6299. doi:10.1038/s41598-017-05871-3. 28740089 PMC5524720

[55] Buchler NE, Louis M. 2008. Molecular titration and ultrasensitivity in regulatory networks. J Mol Biol 384:1106–1119. doi:10.1016/j.jmb.2008.09.079. 18938177

[56] LeRoux M, Culviner PH, Liu YJ, Littlehale ML, Laub MT. 2020. Stress can induce transcription of toxin-antitoxin systems without activating toxin. Mol Cell 79:280–292.E8. doi:10.1016/j.molcel.2020.05.028. 32533919 PMC7368831

[57] Vet S, Vandervelde A, Gelens L. 2019. Excitable dynamics through toxin-induced mRNA cleavage in bacteria. PLoS One 14:e0212288. doi:10.1371/journal.pone.0212288. 30794601 PMC6386449

[58] Tian C, Semsey S, Mitarai N. 2017. Synchronized switching of multiple toxin–antitoxin modules by (p)ppGpp fluctuation. Nucleic Acids Res 45:8180–8189. doi:10.1093/nar/gkx552. 28854732 PMC5737467

[59] Dragoš A, Kiesewalter H, Martin M, Hsu CY, Hartmann R, Wechsler T, Eriksen C, Brix S, Drescher K, Stanley-Wall N, Kümmerli R, Kovács AT. 2018. Division of labor during biofilm matrix production. Curr Biol 28:1903–1913.E5. doi:10.1016/j.cub.2018.04.046. 29887307 PMC6331042

[60] Ogura M. 2016. Post-transcriptionally generated cell heterogeneity regulates biofilm formation in Bacillus subtilis. Genes Cells 21:335–349. doi:10.1111/gtc.12343. 26819068

[61] Thomas P, Terradot G, Danos V, Weiße AY. 2018. Sources, propagation and consequences of stochasticity in cellular growth. Nat Commun 9:4528. doi:10.1038/s41467-018-06912-9. 30375377 PMC6207721

[62] Kleijn IT, Krah LHJ, Hermsen R. 2018. Noise propagation in an integrated model of bacterial gene expression and growth. PLoS Comput Biol 14:e1006386. doi:10.1371/journal.pcbi.1006386. 30289879 PMC6192656

[63] Kiviet DJ, Nghe P, Walker N, Boulineau S, Sunderlikova V, Tans SJ. 2014. Stochasticity of metabolism and growth at the single-cell level. Nature 514:376–379. doi:10.1038/nature13582. 25186725

[64] Chastanet A, Vitkup D, Yuan GC, Norman TM, Liu JS, Losick RM. 2010. Broadly heterogeneous activation of the master regulator for sporulation in Bacillus subtilis. Proc Natl Acad Sci 107:8486–8491. doi:10.1073/pnas.1002499107. 20404177 PMC2889527

[65] Russell JR, Cabeen MT, Wiggins PA, Paulsson J, Losick R. 2017. Noise in a phosphorelay drives stochastic entry into sporulation in Bacillus subtilis. EMBO J 36:2856–2869. doi:10.15252/embj.201796988. 28838935 PMC5623841

[66] Lord ND, Norman TM, Yuan R, Bakshi S, Losick R, Paulsson J. 2019. Stochastic antagonism between two proteins governs a bacterial cell fate switch. Science 366:116–120. doi:10.1126/science.aaw4506. 31604312 PMC7526939

[67] Norman TM, Lord ND, Paulsson J, Losick R. 2013. Memory and modularity in cell-fate decision making. Nature 503:481–486. doi:10.1038/nature12804. 24256735 PMC4019345

[68] Konkol MA, Blair KM, Kearns DB. 2013. Plasmid-encoded ComI inhibits competence in the ancestral 3610 strain of Bacillus subtilis. J Bacteriol 195:4085–4093. doi:10.1128/JB.00696-13. 23836866 PMC3754741

[69] Harwood CR, Cutting SM. 1990. Molecular biological methods for Bacillus. Wiley, Chichester, United Kingdom.

[70] Sambrook J, Russell D. 2001. Molecular cloning: a laboratory manual, 3rd ed. Cold Spring Harbor Laboratory Press, Cold Spring Harbor, NY.

[71] Guérout-Fleury AM, Frandsen N, Stragier P. 1996. Plasmids for ectopic integration in Bacillus subtilis. Gene 180:57–61. doi:10.1016/s0378-1119(96)00404-0. 8973347

[72] Koo BM, Kritikos G, Farelli JD, Todor H, Tong K, Kimsey H, Wapinski I, Galardini M, Cabal A, Peters JM, Hachmann AB, Rudner DZ, Allen KN, Typas A, Gross CA. 2017. Construction and analysis of two genome-scale deletion libraries for Bacillus subtilis. Cell Syst 4:291–305.E7. doi:10.1016/j.cels.2016.12.013. 28189581 PMC5400513

[73] Fujita M, Losick R. 2002. An investigation into the compartmentalization of the sporulation transcription factor σE in Bacillus subtilis. Mol Microbiol 43:27–38. doi:10.1046/j.1365-2958.2002.02732.x. 11849534

[74] Miller JH. 1972. Experiments in molecular genetics. Cold Spring Harbor Laboratory, New York, NY.

[75] de Jong IG, Beilharz K, Kuipers OP, Veening JW. 2011. Live cell imaging of Bacillus subtilis and Streptococcus pneumoniae using automated time-lapse microscopy. J Vis Exp 2011:3145. doi:10.3791/3145. PMC3197447 21841760

[76] Fujita M, Sadaie Y. 1998. Feedback loops involving Spo0A and AbrB in in vitro transcription of the genes involved in the initiation of sporulation in Bacillus subtilis. J Biochem 124:98–104. doi:10.1093/oxfordjournals.jbchem.a022103. 9644251

[77] Weir J, Predich M, Dubnau E, Nair G, Smith I. 1991. Regulation of spo0H, a gene coding for the Bacillus subtilis sigma H factor. J Bacteriol 173:521–529. doi:10.1128/jb.173.2.521-529.1991. 1898930 PMC207041

[78] Narula J, Fujita M, Igoshin OA. 2016. Functional requirements of cellular differentiation: lessons from Bacillus subtilis. Curr Opin Microbiol 34:38–46. doi:10.1016/j.mib.2016.07.011. 27501460

[79] Sekar V, Hageman JH. 1987. Protein turnover and proteolysis during sporulation of Bacillus subtilis. Folia Microbiol (Praha) 32:465–480. doi:10.1007/BF02877199. 3125094

[80] Kov Árová-Kovar K, Egli T. 1998. Growth kinetics of suspended microbial cells: from single-substrate-controlled growth to mixed-substrate kinetics. Microbiol Mol Biol Rev 62:646–666. doi:10.1128/MMBR.62.3.646-666.1998. 9729604 PMC98929

[81] Paintdakhi A, Parry B, Campos M, Irnov I, Elf J, Surovtsev I, Jacobs-Wagner C. 2016. Oufti: an integrated software package for high-accuracy, high-throughput quantitative microscopy analysis. Mol Microbiol 99:767–777. doi:10.1111/mmi.13264. 26538279 PMC4752901

