# Supplementary figures and images for: Bacillus subtilis Histidine Kinase KinC Activates Biofilm Formation by Controlling Heterogeneity of Single-Cell Responses
Source: mBio. 2022 Jan 11;13(1):e01694-21. doi: 10.1128/mbio.01694-21 (PMC8749435; doi:10.1128/mbio.01694-21)

**A**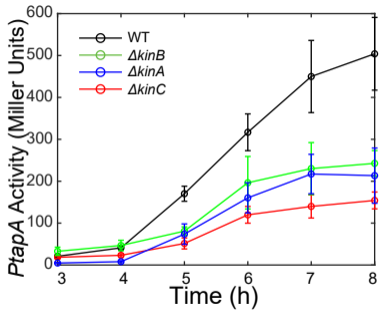**B**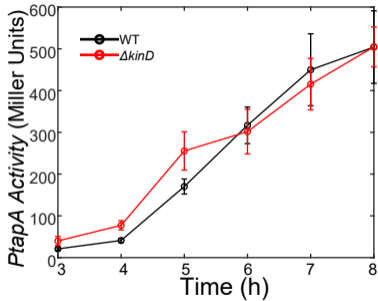

Supplement: FIG S1 [file mbio.01694-21-sf001.pdf]

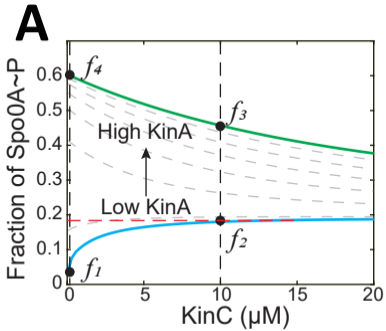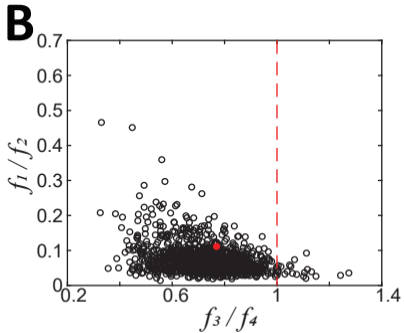

Supplement: FIG S6 [file mbio.01694-21-sf006.pdf]

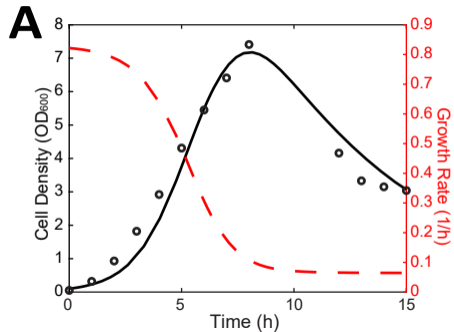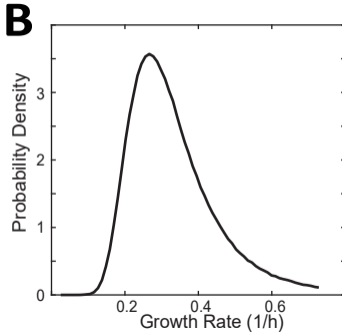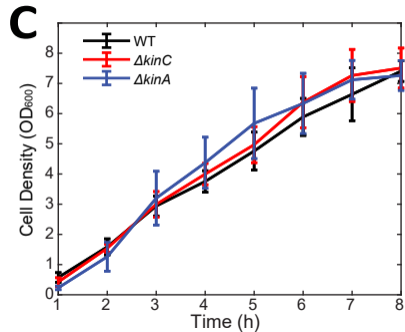

Supplement: FIG S3 [file mbio.01694-21-sf003.pdf]

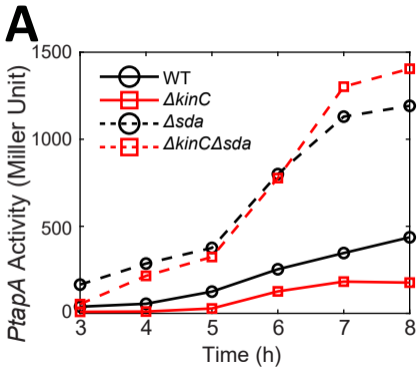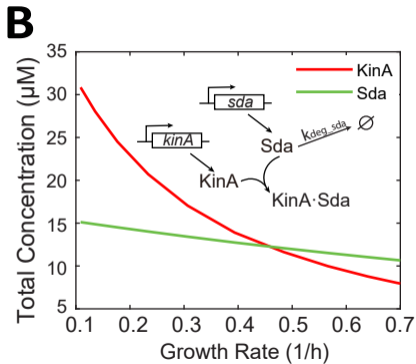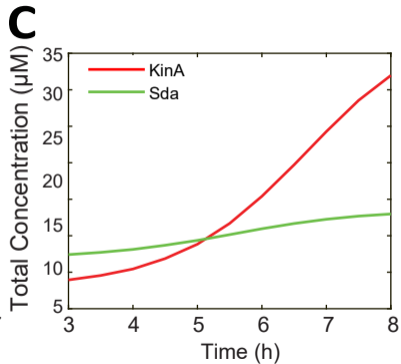

Supplement: FIG S2 [file mbio.01694-21-sf002.pdf]

**A**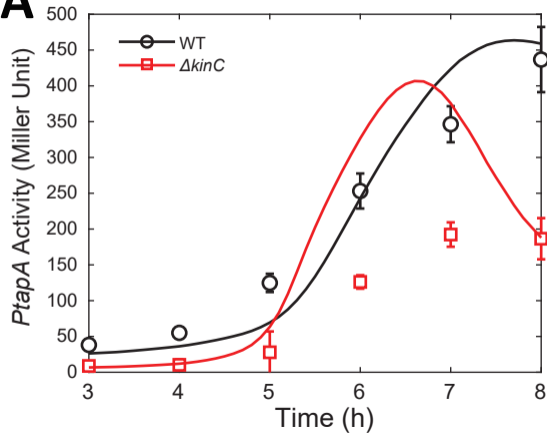**B**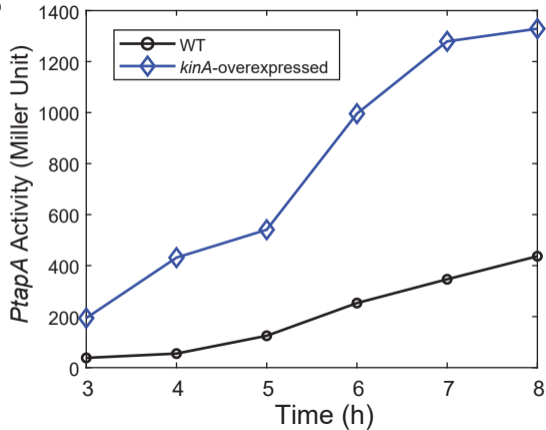

Supplement: FIG S4 [file mbio.01694-21-sf004.pdf]

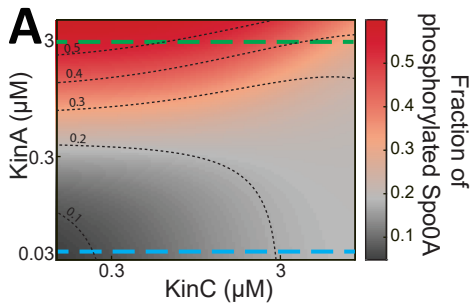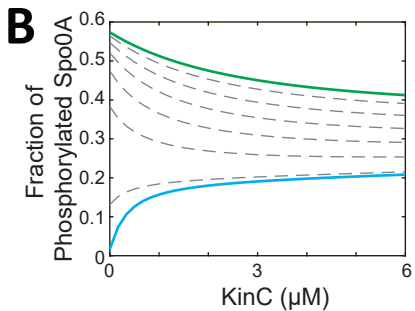

Supplement: FIG S5 [file mbio.01694-21-sf005.pdf]
