# Supplementary material for: Bacillus subtilis Histidine Kinase KinC Activates Biofilm Formation by Controlling Heterogeneity of Single-Cell Responses
Source: mBio. 2022 Jan 11;13(1):e01694-21. doi: 10.1128/mbio.01694-21 (PMC8749435; doi:10.1128/mbio.01694-21)
Supplement: TABLE S2 [file mbio.01694-21-st002.pdf]

Table S2 List of strains and plasmids used in this work

***B. subtilis* strains used for microscopy**

Name	Genotype and relevant features	Source/reference	Construction*
**MF8771**	*thrC::PtapA-gfp erm comI*^{Q12L}	This study	MF5680 → DK1042
**MF8957**	*ΔkinC thrC::PtapA-gfp erm comI*^{Q12L}	This study	MF5680 → MF8068
**MF11174**	*kinAΩkinA-gfp kan amyE::PtapA-mCherry spc*^{Q12L}	This study	MF11054 → MF7710
**MF11192**	*ΔkinC::erm kinAΩkinA-gfp kan amyE::PtapA-mCherry spc comI*^{Q12L}	This study	BKE14490 → MF11174

***B. subtilis* strains used for β-galactosidase assay**

Name	Genotype and relevant features	Source/reference	Construction*
**MF5616**	*thrC::PspoIIG-lacZ erm comI*^{Q12L}	This study	MF750 → DK1042
**MF5763**	*thrC::PtapA-lacZ erm comI*^{Q12L}	[1]	
**MF6964**	*thrC::PtapA-lacZ erm ΔkinC::cm kinAΩPhy-spank-kinA spc comI*^{Q12L}	This study	MF5652 → MF5697
**MF9089**	*Δsda::erm amyE::PtapA-lacZ cm comI*^{Q12L}	This study	MF6110 → MF7463
**MF9140**	*Δsda::erm ΔkinC amyE::PtapA-lacZ cm comI*^{Q12L}	This study	MF6110 → MF9134
**MF9162**	*ΔkinA thrC::PtapA-lacZ erm comI*^{Q12L}	This study	MF5652 → MF8816
**MF9164**	*ΔkinC thrC::PtapA-lacZ erm comI*^{Q12L}	This study	MF5652 → MF8068
**MF9168**	*ΔkinA thrC::PspoIIG-lacZ erm comI*^{Q12L}	This study	MF750 → MF8816
**MF9170**	*ΔkinC thrC::PspoIIG-lacZ erm comI*^{Q12L}	This study	MF750 → MF8068
**MF9163**	*ΔkinB thrC::PtapA-lacZ erm comI*^{Q12L}	This study	MF5652 → MF8067
**MF11241**	*ΔkinD thrC::PtapA-lacZ erm comI*^{Q12L}	This study	MF5652 → MF6441

***B. subtilis* strains used for sporulation efficiency**

Name	Genotype and relevant features	Source/reference	Construction*
**DK1042**	*comI*^{Q12L} undomesticated competent wild type strain	[2]	
**MF8068**	*ΔkinC comI*^{Q12L}	This study	pDR244 → MF6712
**MF8816**	*ΔkinA comI*^{Q12L}	This study	pDR244 → MF8066

***B. subtilis* strains for strain construction**

Name	Genotype and relevant features	Source/reference	Construction*
**DK1042**	*comI*^{Q12L} undomesticated competent wild type strain	[2]	
**PY79**	prototroph	[3]	
**MF1887**	*kinAΩPhy-spank-kinA spc*	[4]	
**MF5652**	*thrC::PtapA-lacZ erm*	This study	pMF712 → PY79
**MF750**	*thrC::PspoIIG-lacZ erm*	[5]	
**MF929**	*kinAΩkinA-gfp kan*	[4]	
**MF11054**	*amyE::PtapA-mCherry spc*	This study	pMF1130 → PY79
**MF5680**	*thrC::PtapA-gfp erm*	This study	pMF719 → PY79
**BKE13990**	*ΔkinA::erm trpC2*	BGSC†	
**BKE31450**	*ΔkinB::erm trpC2*	BGSC†	
**BKE14490**	*ΔkinC::erm trpC2*	BGSC†	
**BKE13660**	*ΔkinD::erm trpC2*	BGSC†	
**BKE25690**	*Δsda::erm trpC2*	BGSC†	
**MF8066**	*ΔkinA::erm comI*^{Q12L}	This study	BKE13990 → DK1042
**MF6711**	*ΔkinB::erm comI*^{Q12L}	This study	BKE31450 → DK1042
**MF6712**	*ΔkinC::erm comI*^{Q12L}	This study	BKE14490 → DK1042
**MF6439**	*ΔkinD::erm comI*^{Q12L}	This study	BKE13660 → DK1042
**MF9099**	*Δsda::erm comI*^{Q12L}	This study	BKE25690 → DK1042
**MF8816**	*ΔkinA comI*^{Q12L}	This study	pMF244 → MF8066
**MF8067**	*ΔkinB comI*^{Q12L}	This study	pMF244 → MF6711
**MF8068**	*ΔkinC comI*^{Q12L}	This study	pMF244 → MF6712
**MF6441**	*ΔkinD comI*^{Q12L}	This study	pMF244 → MF6439
**MF7710**	*kinAΩKinA-gfp kan comI*^{Q12L}	This study	MF929 → DK1042
**MF5697**	*ΔkinB::kan ΔkinC::cm kinAΩPhy-spank-kinA spc comI*^{Q12L}	This study	MF1847 → MF5695
**MF5695**	*ΔkinC::cm kinAΩPhy-spank-kinA spc comI*^{Q12L}	This study	MF1845 → MF5611
**MF5611**	*kinAΩPhy-spank-kinA spc comI*^{Q12L}	This study	MF1887 → DK1042
**MF1847**	*ΔkinB::kan*	[6]	
**MF1845**	*ΔkinC::cm*	[7]	

Plasmids

Name	Genotype and relevant features	Source/reference
**pDR244**	Cre-expressing plasmid containing a spectinomycin resistance gene and a temperature-sensitive replication origin in *B. subtilis*	[8]
**pMF712**	*thrC::PtapA-lacZ erm*	[1]
**pMF719**	*thrC::PtapA-gfp erm*	This study
**pMF1130**	*amyE::PtapA-mCherry spc*	This study

*Arrows indicate transformation and point from donor DNA to recipient strain
†Bacillus Genetic Stock Center

References

[1] S. N. Devi, M. Vishnoi, B. Kiehler, L. Haggett, and M. Fujita, “In vivo functional characterization of the transmembrane histidine kinase KinC in Bacillus subtilis,” *Microbiology,*, vol. 161, no. 5, pp. 1092–1104, 2015.

[2] M. A. Konkol, K. M. Blair, and D. B. Kearns, “Plasmid-Encoded ComI Inhibits Competence in the Ancestral 3610 Strain of Bacillus subtilis,” *Journal of Bacteriology*, vol. 195, pp. 4085–4093, Sept. 2013.

[3] P. Youngman, J. B. Perkins, and R. Losick, “Construction of a cloning site near one end of tn917 into which foreign dna may be inserted without affecting transposition in bacillus subtilis or expression of the transposon-borne erm gene,” *Plasmid*, vol. 12, no. 1, pp. 1–9, 1984.

[4] M. Fujita and R. Losick, “Evidence that entry into sporulation in Bacillus subtilis is governed by a gradual increase in the level and activity of the master regulator Spo0A,” *Genes & development*, vol. 19, pp. 2236–2244, 2005.

[5] P. Eswaramoorthy, T. Guo, and M. Fujita, “In vivo domain-based functional analysis of the major sporulation sensor kinase, KinA, in Bacillus subtilis,” *Journal of Bacteriology*, vol. 191, pp. 5358–5368, Sept. 2009.

[6] S. Tojo, K. Hirooka, and Y. Fujita, “Expression of kina and kinb of bacillus subtilis, necessary for sporulation initiation, is under positive stringent transcription control,” *Journal of bacteriology*, vol. 195, no. 8, pp. 1656–1665, 2013.

[7] A. V. Banse, E. C. Hobbs, and R. Losick, “Phosphorylation of spo0a by the histidine kinase kind requires the lipoprotein med in bacillus subtilis,” *Journal of bacteriology*, vol. 193, no. 15, pp. 3949–3955, 2011.

[8] B.-M. Koo, G. Kritikos, J. D. Farelli, H. Todor, K. Tong, H. Kimsey, I. Wapinski, M. Galardini, A. Cabal, J. M. Peters, A.-B. Hachmann, D. Z. Rudner, K. N. Allen, A. Typas, and C. A. Gross, “Construction and Analysis of Two Genome-Scale Deletion Libraries for Bacillus subtilis,” *Cell Systems*, vol. 4, pp. 291–305.e7, Mar. 2017.
