# Supplementary material for: Bacillus subtilis Histidine Kinase KinC Activates Biofilm Formation by Controlling Heterogeneity of Single-Cell Responses
Source: mBio. 2022 Jan 11;13(1):e01694-21. doi: 10.1128/mbio.01694-21 (PMC8749435; doi:10.1128/mbio.01694-21)
Supplement: TABLE S1 [file mbio.01694-21-st001.pdf]

Table S1 Parameters used in the mathematical models

Post-translational reactions and parameters of the phosphorelay model	
Reaction	Parameters
$\mathrm{KinA} \underset{k_{a2}}{\overset{k_{a1}}{\rightleftharpoons}} \mathrm{KinA{\sim}P}$	k_{a1} =12 h^{-1},k_{a2} =2 h^{-1}
$\mathrm{KinC} \underset{k_{c2}}{\overset{k_{c1}}{\rightleftharpoons}} \mathrm{KinC{\sim}P}$	k_{c1} =12 h^{-1},k_{c2} =2 h^{-1}
$\mathrm{KinA{\sim}P} + \mathrm{Spo0F} \underset{k_{a3}}{\overset{k_b}{\rightleftharpoons}} \mathrm{KinA \cdot Spo0F{\sim}P} \underset{k_b}{\overset{k_{a4}}{\rightleftharpoons}} \mathrm{KinA} + \mathrm{Spo0F{\sim}P}$	k_b =500 µ$M^{-1}h^{-1}$<br>k_{a3} =500 h^{-1}, k_{a4} =200 h^{-1}
$\mathrm{KinA} + \mathrm{Sda} \underset{k_s}{\overset{k_b}{\rightleftharpoons}} \mathrm{KinA \cdot Sda}$	k_s =5 h^{-1}
$\mathrm{KinA} + \mathrm{Spo0F} \underset{k_f}{\overset{k_b}{\rightleftharpoons}} \mathrm{KinA \cdot Spo0F}$	k_f =100 h^{-1}
$\mathrm{KinC{\sim}P} + \mathrm{Spo0F} \underset{k_{c3}}{\overset{k_b}{\rightleftharpoons}} \mathrm{KinC \cdot Spo0F{\sim}P} \underset{k_b}{\overset{k_{c4}}{\rightleftharpoons}} \mathrm{KinC} + \mathrm{Spo0F{\sim}P}$	k_{c3} =700 h^{-1}, k_{c4} =10 h^{-1}
$\mathrm{Spo0F{\sim}P} + \mathrm{Spo0B} \underset{k_1}{\overset{k_b}{\rightleftharpoons}} \mathrm{Spo0F \cdot Spo0B{\sim}P} \underset{k_b}{\overset{k_2}{\rightleftharpoons}} \mathrm{Spo0F} + \mathrm{Spo0B{\sim}P}$	k_1 =200 h^{-1}, k_2 =800 h^{-1}
$\mathrm{Spo0B{\sim}P} + \mathrm{Spo0A} \underset{k_3}{\overset{k_b}{\rightleftharpoons}} \mathrm{Spo0B \cdot Spo0A{\sim}P} \underset{k_b}{\overset{k_4}{\rightleftharpoons}} \mathrm{Spo0B} + \mathrm{Spo0A{\sim}P}$	k_3 =200 h^{-1}, k_4 =800 h^{-1}
$\mathrm{Spo0A{\sim}P} + \mathrm{Spo0E} \underset{k_5}{\overset{k_b}{\rightleftharpoons}} \mathrm{Spo0E \cdot Spo0A{\sim}P} \overset{k_6}{\longrightarrow} \mathrm{Spo0E} + \mathrm{Spo0A}$	k_5 =200 h^{-1}, k_6 =10 h^{-1}

Production rates of species in the phosphorelay model			
Species	Production rate	Parameters	Gene positions
KinA	v_a	v_a =4 µM·h^{-1}	0.70
KinC	v_c	v_c =2.7 µM·h^{-1}	0.72
Spo0F	v_f	v_f =2 µM·h^{-1}	0.19
Spo0B	v_b	v_b =2 µM·h^{-1}	0.65
Spo0A	$v_0 + v_1 \frac{[\mathrm{Spo0A{\sim}P}]^2}{[\mathrm{Spo0A{\sim}P}]^2 + K_a^2}$	v_0 =0.4 µM·h^{-1},<br>v_1 =0.5µM·h^{-1} K_a =0.05 µM	0.80
Spo0E	v_e	v_e =0.2 µM·h^{-1}	0.68
Sda	v_s	v_s =57 µM·h^{-1}	0.74
TapA	$\frac{[\mathrm{Spo0A{\sim}P}]^{n_t}}{[\mathrm{Spo0A{\sim}P}]^{n_t} + K_t^{n_t}}$	n_t =0.98*, K_t =8.25 µM·h^{-1}*<br>n_t =0.15†, K_t =4 µM·h^{-1}†	0.43§
TapA‡	$\frac{[\mathrm{Spo0A{\sim}P}]^{n_{t1}}}{[\mathrm{Spo0A{\sim}P}]^{n_{t1}} + K_{t1}^{n_{t1}}} \frac{K_{t2}^{n_{t2}}}{[\mathrm{Spo0A{\sim}P}]^{n_{t2}} + K_{t2}^{n_{t2}}}$	n_{t1} =1.15*, K_{t1} =1.51 µM·h^{-1}*<br>n_{t2} =8*, K_{t2} =1.66 µM·h^{-1}*	0.43§
SpoIIG	$\frac{[\mathrm{Spo0A{\sim}P}]^{n_g}}{[\mathrm{Spo0A{\sim}P}]^{n_g} + K_g^{n_g}}$	K_g =2.7 µM*, n_g =4<br>K_g =1.2 µM†, n_g =4	0.43§

Parameters of the model for the growth dynamics¶			
k_g	1.90 h^{-1}	k_d	0.612 h^{-1}
ψ	0.3	h_1	1.88
h_2	2.89	K_1	1.21
K_2	0.148	γ	0.11

*Fitted parameters

†Different parameters using in the model of the noise in growth rate

‡Alternative model that includes the repression of *tapA* expression by high Spo0A~P levels

§The position of *thrC* where *PtapA-lacZ* and *PspoIIG-lacZ* were inserted

¶In the model of growth dynamics, the cell density (which is represented by OD) and nutrient level are considered as dimensionless variables, so the parameters do not have units except the time unit.
