# Supplementary material for: Bacillus subtilis Histidine Kinase KinC Activates Biofilm Formation by Controlling Heterogeneity of Single-Cell Responses
Source: mBio. 2022 Jan 11;13(1):e01694-21. doi: 10.1128/mbio.01694-21 (PMC8749435; doi:10.1128/mbio.01694-21)
Supplement: TABLE S3 [file mbio.01694-21-st003.pdf]

Table S3 The differential equations of the phosphorelay model

$$\frac{d}{dt}[\text{KinA}] = v_a F(\mu) + k_{a4}[\text{KinA}\cdot\text{Spo0F}{\sim}\text{P}] - k_b[\text{KinA}][\text{Spo0F}{\sim}\text{P}] - k_{a1}[\text{KinA}] + k_{a2}[\text{KinA}{\sim}\text{P}] - k_b[\text{Spo0F}][\text{KinA}] + k_f[\text{Spo0F}\cdot\text{KinA}] - k_b[\text{KinA}][\text{Sda}] + k_s[\text{KinA}\cdot\text{Sda}] - k_{deg}[\text{KinA}]$$

$$\frac{d}{dt}[\text{KinA}\cdot\text{Sda}] = k_b[\text{KinA}][\text{Sda}] - k_s[\text{KinA}\cdot\text{Sda}] - k_{deg}[\text{KinA}\cdot\text{Sda}]$$

$$\frac{d}{dt}[\text{KinA}{\sim}\text{P}] = -k_b[\text{KinA}{\sim}\text{P}][\text{Spo0F}] + k_{a3}[\text{KinA}\cdot\text{Spo0F}{\sim}\text{P}] + k_{a1}[\text{KinA}] - k_{a2}[\text{KinA}{\sim}\text{P}] - k_{deg}[\text{KinA}{\sim}\text{P}]$$

$$\frac{d}{dt}[\text{KinA}\cdot\text{Spo0F}{\sim}\text{P}] = k_b[\text{KinA}{\sim}\text{P}][\text{Spo0F}] - k_{a3}[\text{KinA}\cdot\text{Spo0F}{\sim}\text{P}] - k_{a4}[\text{KinA}\cdot\text{Spo0F}{\sim}\text{P}] + k_b[\text{KinA}][\text{Spo0F}{\sim}\text{P}] - k_{deg}[\text{KinA}\cdot\text{Spo0F}{\sim}\text{P}]$$

$$\frac{d}{dt}[\text{KinC}] = v_c F(\mu) + k_{c4}[\text{KinC}\cdot\text{Spo0F}{\sim}\text{P}] - k_b[\text{KinC}][\text{Spo0F}{\sim}\text{P}] - k_{c1}[\text{KinC}] + k_{c2}[\text{KinC}{\sim}\text{P}] - k_{deg}[\text{KinC}]$$

$$\frac{d}{dt}[\text{KinC}{\sim}\text{P}] = -k_b[\text{KinC}{\sim}\text{P}][\text{Spo0F}] + k_{c3}[\text{KinC}\cdot\text{Spo0F}{\sim}\text{P}] + k_{c1}[\text{KinC}] - k_{c2}[\text{KinC}{\sim}\text{P}] - k_{deg}[\text{KinC}{\sim}\text{P}]$$

$$\frac{d}{dt}[\text{KinC}\cdot\text{Spo0F}{\sim}\text{P}] = k_b[\text{KinC}{\sim}\text{P}][\text{Spo0F}] - k_{c3}[\text{KinC}\cdot\text{Spo0F}{\sim}\text{P}] - k_{c4}[\text{KinC}\cdot\text{Spo0F}{\sim}\text{P}] + k_b[\text{KinC}][\text{Spo0F}{\sim}\text{P}] - k_{deg}[\text{KinC}\cdot\text{Spo0F}{\sim}\text{P}]$$

$$\frac{d}{dt}[\text{Sda}] = v_s F(\mu) - k_b[\text{KinA}][\text{Sda}][+k_s[\text{KinA}\cdot\text{Sda}] - k_{degs}[\text{Sda}]$$

$$\frac{d}{dt}[\text{Spo0A}] = (v_0 + v_1\frac{[\text{Spo0A}{\sim}\text{P}]^2}{[\text{Spo0A}{\sim}\text{P}]^2+K_a^2})F(\mu) - k_b[\text{Spo0A}\cdot\text{Spo0B}{\sim}\text{P}] + k_3[\text{Spo0B}\cdot\text{Spo0A}{\sim}\text{P}] + k_6[\text{Spo0E}\cdot\text{Spo0A}{\sim}\text{P}] - k_{deg}[\text{Spo0A}]$$

$$\frac{d}{dt}[\text{Spo0A}{\sim}\text{P}] = k_4[\text{Spo0B}\cdot\text{Spo0A}{\sim}\text{P}] - k_b[\text{Spo0B}\cdot\text{Spo0A}{\sim}\text{P}] - k_b[\text{Spo0E}\cdot\text{Spo0A}{\sim}\text{P}] + k_5[\text{Spo0E}\cdot\text{Spo0A}{\sim}\text{P}] - k_{deg}[\text{Spo0A}{\sim}\text{P}]$$

$$\frac{d}{dt}[\text{Spo0E}\cdot\text{Spo0A}{\sim}\text{P}] = k_b[\text{Spo0E}\cdot\text{Spo0A}{\sim}\text{P}] - k_5[\text{Spo0E}\cdot\text{Spo0A}{\sim}\text{P}] - k_6[\text{Spo0E}\cdot\text{Spo0A}{\sim}\text{P}] - k_{deg}[\text{Spo0E}\cdot\text{Spo0A}{\sim}\text{P}]$$

$$\frac{d}{dt}[\text{Spo0B}] = v_b F(\mu) + k_4[\text{Spo0B}\cdot\text{Spo0A}{\sim}\text{P}] - k_b[\text{Spo0B}\cdot\text{Spo0A}{\sim}\text{P}] - k_b[\text{Spo0F}{\sim}\text{P}][\text{Spo0B}] + k_3[\text{Spo0F}\cdot\text{Spo0B}{\sim}\text{P}] - k_{deg}[\text{Spo0B}]$$

$$\frac{d}{dt}[\text{Spo0B}{\sim}\text{P}] = -k_b[\text{Spo0A}\cdot\text{Spo0B}{\sim}\text{P}] + k_3[\text{Spo0B}\cdot\text{Spo0A}{\sim}\text{P}] + k_4[\text{Spo0F}\cdot\text{Spo0B}{\sim}\text{P}] - k_b[\text{Spo0F}][\text{Spo0B}{\sim}\text{P}] - k_{deg}[\text{Spo0B}{\sim}\text{P}]$$

$$\frac{d}{dt}[\text{Spo0B}\cdot\text{Spo0A}{\sim}\text{P}] = k_b[\text{Spo0A}\cdot\text{Spo0B}{\sim}\text{P}] - k_3[\text{Spo0B}\cdot\text{Spo0A}{\sim}\text{P}] - k_4[\text{Spo0B}\cdot\text{Spo0A}{\sim}\text{P}] + k_b[\text{Spo0B}\cdot\text{Spo0A}{\sim}\text{P}] - k_{deg}[\text{Spo0B}\cdot\text{Spo0A}{\sim}\text{P}]$$

$$\frac{d}{dt}[\text{Spo0E}] = v_e F(\mu) - k_b[\text{Spo0E}\cdot\text{Spo0A}{\sim}\text{P}] + k_5[\text{Spo0E}\cdot\text{Spo0A}{\sim}\text{P}] + k_6[\text{Spo0E}\cdot\text{Spo0A}{\sim}\text{P}] - k_{deg}[\text{Spo0E}]$$

$$\frac{d}{dt}[\text{Spo0F}] = v_f F(\mu) + k_2[\text{Spo0F}\cdot\text{Spo0B}{\sim}\text{P}] - k_b[\text{Spo0F}][\text{Spo0B}{\sim}\text{P}] - k_b[\text{KinA}{\sim}\text{P}][\text{Spo0F}] + k_{a3}[\text{KinA}\cdot\text{Spo0F}{\sim}\text{P}] - k_b[\text{KinC}{\sim}\text{P}][\text{Spo0F}] + k_{c3}[\text{KinC}\cdot\text{Spo0F}{\sim}\text{P}] - k_b[\text{Spo0F}][\text{KinA}] + k_f[\text{Spo0F}\cdot\text{KinA}] - k_{deg}[\text{Spo0F}]$$

$$\frac{d}{dt}[\text{Spo0F}\cdot\text{KinA}] = k_b[\text{Spo0F}][\text{KinA}] - k_f[\text{Spo0F}\cdot\text{KinA}] - k_{deg}[\text{Spo0F}\cdot\text{KinA}]$$

$$\frac{d}{dt}[\text{Spo0F}{\sim}\text{P}] = -k_b[\text{Spo0F}{\sim}\text{P}][\text{Spo0B}] + k_1[\text{Spo0F}\cdot\text{Spo0B}{\sim}\text{P}] + k_{a4}[\text{KinA}\cdot\text{Spo0F}{\sim}\text{P}] - k_b[\text{KinA}][\text{Spo0F}{\sim}\text{P}] + k_{c4}[\text{KinC}\cdot\text{Spo0F}{\sim}\text{P}] - k_b[\text{KinC}][\text{Spo0F}{\sim}\text{P}] - k_{deg}[\text{Spo0F}{\sim}\text{P}]$$

$$\frac{d}{dt}[\text{Spo0F}\cdot\text{Spo0B}{\sim}\text{P}] = k_b[\text{Spo0F}{\sim}\text{P}][\text{Spo0B}] - k_1[\text{Spo0F}\cdot\text{Spo0B}{\sim}\text{P}] - k_2[\text{Spo0F}\cdot\text{Spo0B}{\sim}\text{P}] + k_b[\text{Spo0F}][\text{Spo0B}{\sim}\text{P}] - k_{deg}[\text{Spo0F}\cdot\text{Spo0B}{\sim}\text{P}]$$

$F(\mu)$ represents the effect of growth rate(μ) on the production rate of the proteins.
